# Anticancer Ruthenium(III) Complexes and Ru(III)-Containing Nanoformulations: An Update on the Mechanism of Action and Biological Activity

**DOI:** 10.3390/ph12040146

**Published:** 2019-09-26

**Authors:** Claudia Riccardi, Domenica Musumeci, Marco Trifuoggi, Carlo Irace, Luigi Paduano, Daniela Montesarchio

**Affiliations:** 1 Department of Chemical Sciences, University of Naples Federico II, Via Cintia 21, I-80126 Naples, Italy; claudia.riccardi@unina.it (C.R.); domenica.musumeci@unina.it (D.M.); marco.trifuoggi@unina.it (M.T.); luigi.paduano@unina.it (L.P.); 2 Department of Pharmacy, University of Naples Federico II, Via D. Montesano 49, I-80131 Naples, Italy; carlo.irace@unina.it

**Keywords:** Ruthenium(III) complexes, nanocarriers, nanoaggregates, drug delivery, anticancer therapy, preclinical evaluation

## Abstract

The great advances in the studies on metal complexes for the treatment of different cancer forms, starting from the pioneering works on platinum derivatives, have fostered an increasingly growing interest in their properties and biomedical applications. Among the various metal-containing drugs investigated thus far, ruthenium(III) complexes have emerged for their selective cytotoxic activity in vitro and promising anticancer properties in vivo, also leading to a few candidates in advanced clinical trials. Aiming at addressing the solubility, stability and cellular uptake issues of low molecular weight Ru(III)-based compounds, some research groups have proposed the development of suitable drug delivery systems (e.g., taking advantage of nanoparticles, liposomes, etc.) able to enhance their activity compared to the naked drugs. This review highlights the unique role of Ru(III) complexes in the current panorama of anticancer agents, with particular emphasis on Ru-containing nanoformulations based on the incorporation of the Ru(III) complexes into suitable nanocarriers in order to enhance their bioavailability and pharmacokinetic properties. Preclinical evaluation of these nanoaggregates is discussed with a special focus on the investigation of their mechanism of action at a molecular level, highlighting their pharmacological potential in tumour disease models and value for biomedical applications.

## 1. Introduction

### 1.1. From Platinum(II) to Ruthenium(III)-based Complexes: The Importance of Nanoformulations in Metallo Drug Delivery

The interest in metal-based complexes for the treatment of cancer started with the serendipitous discovery of the anti-tumour properties of *cis*-diamminedichloroplatinum(II) (*c*DDP or cisplatin) in the early 1960s [1,2]. Approved in 1978 by the Food and Drug Administration [3], cisplatin is used in the treatment of a broad spectrum of human cancer malignancies either as single agent (cervical, bladder, head, and neck cancer) or in combination treatments (testicular, bladder, head and neck cancer) [4]. The cytotoxicity of cisplatin is due to its interaction with DNA, forming adducts preferentially with adjacent guanines, thus interfering with replication and transcription processes and ultimately triggering apoptosis, as main cell death pathway [5,6,7,8]. Unfortunately, dose-limiting side effects (nephrotoxicity, ototoxicity and peripheral neurotoxicity) and its intrinsic or acquired resistance hindered its widespread use [4,9,10], stimulating the search for new, safer metal-based anticancer agents.

Second- and also third-generation Pt(II) complexes have been developed over the last 50 years, with about more than 20 Pt(II)-based compounds entered in clinical trials. Of these, only two (carboplatin [11] and oxaliplatin [12]) obtained international marketing approval, respectively, in 1992 and 2002, and three others (nedaplatin, lobaplatin and heptaplatin) were approved only in individual countries (Japan, China and Korea, respectively) [13,14,15].

Afterwards, novel anticancer agents based on metals different from platinum were developed, as extensively reported in several review articles [16,17,18,19,20,21,22,23]. Among all the metal derivatives thus far investigated, ruthenium complexes in the +2 and +3 oxidation states gained increasing attention as valuable alternatives to Pt(II)-based ones [24,25,26,27,28,29,30,31,32,33].

However, clinical application of metallodrugs, when administered via conventional intravenous methods, has been hampered by their limited aqueous solubility and short in vivo half-lives, resulting in inadequate bioavailability and low accumulation in the tumour masses. Thus, several approaches have been proposed to address these issues, particularly exploiting nanotechnology-based strategies [34,35,36,37]. The main advantages of using nanosystems for drug delivery include their high stability, notable loading capacity and possibility to achieve controlled or sustained drug release. This approach can significantly increase the circulation time in the body, limit the susceptibility to chemical and/or enzymatic degradation, target specific tumour sites and reduce the toxic side effects associated with drug administration [28,37,38,39,40,41,42].

As far as Ru(II)-based complexes are concerned, their preparation, encapsulation into different nanosystems and studies on their mechanism of action have been comprehensively described in recent overviews [43,44].

In this review we report the state-of-the-art on anticancer Ru(III)-based complexes including the ad hoc designed nanoformulations to incorporate and deliver these antiproliferative agents. Particular emphasis is given to the emerging strategies to facilitate the application of Ru(III)-based drugs in vivo, aimed at enhancing their solubility and bioavailability, as well as improving their delivery to cancer cells. In addition, the biological activity of these Ru(III)-containing nanosystems and, where known, their mechanism of action is discussed, focusing on the contribution of our group in this field and the most recent literature examples.

### 1.2. Anticancer Activity and Mechanism of Action of the Lead Low Molecular Weight Ru(III)-Based Compounds

**Figure 1:**
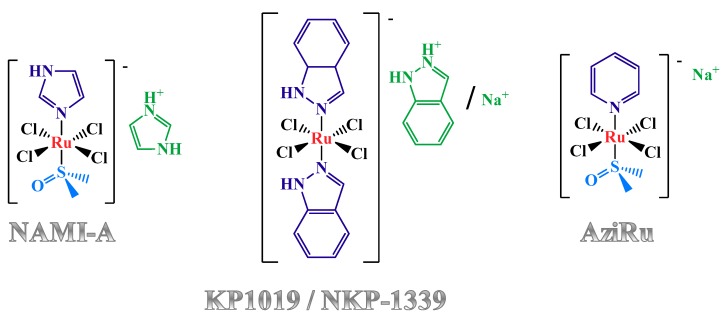
Chemical structures of NAMI-A, KP1019, NKP-1339 and AziRu. Here, ruthenium ions are indicated in red, the counterions in green, while the N- and S-donor ligands in dark and light blue, respectively.

To the best of our knowledge, three Ru(III)-containing compounds entered in clinical trials: NAMI-A [45,46], KP1019 [47,48,49,50,51,52,53], as well as its sodium salt analogue named IT-139, formerly known as NKP-1339 [54,55,56,57,58]. Despite their structural similarities (Figure 1), these complexes dramatically differ in their bioactivity [59]. In fact, preclinical studies demonstrated that NAMI-A was essentially inactive against primary tumours but proved to specifically affect tumour metastases, preventing their development and growth [45,46]. In contrast, KP1019 exhibited marked cytotoxic activity in vitro in cisplatin-resistant human colon carcinoma cell lines, as well as significant anti-tumour effects in vivo against a wide variety of tumour xenografts through induction of apoptosis [47,48,49,50,51,52,53]. NKP-1339 is the most recent compound entered in clinical trials against solid cancer forms, showing a manageable safety profile with absence of neurotoxicity and dose-limiting haematological toxicity [54,55,56,57,58].

Many preclinical studies have investigated the mechanism of action of these complexes. A large body of evidence has been gathered proving that these Ru(III) complexes are able to interact with plasma proteins—particularly with serum albumin [60,61,62,63,64] and transferrin [65,66,67,68,69]—and/or bind nucleic acids [70,71,72,73]. The extensive binding to serum proteins for KP1019 and NAMI-A was also reflected by their low distribution volume in clinical phase I evaluation [45,51].

As concerns the interaction with cancer cells, X-ray fluorescence imaging measurements revealed a wide intracellular distribution of KP1019 both in cytosol and in the nuclear region after treatment; conversely, following NAMI-A administration in vitro, ruthenium was not detected inside the cells, suggesting its strong interaction with cell membranes as well as alternative mechanisms of action [74].

Stimulated by the positive impact of Ru(III) complexes in the panorama of known anticancer agents, Walsby et al. [75,76] as well as our group [77] independently and almost simultaneously described a NAMI-A-like complex carrying a pyridine residue in place of the imidazole ligand, and sodium replacing imidazolium as the counterion (Figure 1, right). This novel compound, called NAMI-Pyr by the first research group and AziRu by the second one, overall proved to be poorly cytotoxic, similarly to the parent NAMI-A, however showing IC_50_ values half that of NAMI-A on human breast MCF-7 and cervical HeLa cancer cell lines (Table 1) [77,78,79].

The overall higher cytotoxicity observed for this pyridine-containing Ru complex vs. NAMI-A was explained by the chemical properties of the nitrogen ligand, which compared to imidazole confers higher lipophilicity and, thus, expectedly improved cellular uptake [75,80,81,82].

Analogously to its congeners, AziRu proved to bind both nucleic acids and proteins [83,84,85,86]. Detailed spectroscopic and mass spectrometric investigations showed AziRu to be more reactive towards DNA model systems—both single stranded and duplex oligonucleotides—than NAMI-A [85]. Moreover, its capability to interact with model proteins—such as bovine pancreatic ribonuclease A (RNase A) [84] and hen egg white lysozyme (HEWL) [83]—was investigated by X-ray crystallography and Raman microscopy studies. In both model proteins, crystal structure analysis indicated that the protein conformation was not dramatically affected by Ru complexation and that the metal lost all its original ligands upon binding [83,84]. In particular, experiments with HEWL protein also underlined the ability of AziRu, upon aging, to form polyoxo species containing Ru-O-Ru bond [83,87].

More recently, studies aimed at identifying AziRu release conditions from bound proteins were carried out, using a Raman-assisted crystallographic approach to investigate the AziRu/HEWL complex as a case study. Crystallography was used to identify the protein structural changes and metal release occurring upon Ru^III^ → Ru^II^ reduction by alternative exposure to different reducing agents, while Raman microscopy on protein crystals allowed identifying the spectral changes attributable to the reduction of Ru(III) bound to the protein [86]. These studies clearly indicated a Ru reduction in the protein complex, followed by a Ru release mechanism dependent on the reducing agent: reduction with hydrazine produced a native-like lysozyme crystal with a Raman spectrum identical to the wild-type protein, suggesting the complete Ru release from the protein upon reduction. In contrast, the Raman analysis on AziRu/HEWL crystals treated with ascorbate suggested that in this case the Ru^III^ → Ru^II^ reduction occurred without Ru release. The observed differences in the hydrazine- and ascorbate-induced action indicated a two-step Ru reduction-release mechanism [86]. To better understand this process, a pH-dependent, spectroelectrochemical surface-enhanced Raman scattering (SERS) study was also performed on AziRu-functionalized Au electrodes as a surrogate and simple model system mimicking the anticancer Ru(III)-based drugs. This SERS study allowed determining a pKa of 6.0 ± 0.4 for the aquated AziRu complex in the Ru^III^ state, which corresponds to the pH values characteristic of the external microenvironment of most cancer cells, significantly differing from healthy ones. These experiments also indicated a dramatic shift of the redox potential (E_0_) by > 600 mV for the aquated AziRu species toward more positive potentials upon acidification, suggesting a selective AziRu reduction in cancer environments but not in normal ones [86].

### 1.3. Nucleolipid and Aminoacyl Lipid-based Structures Incorporating AziRu, a NAMI-A-Like Ruthenium Compound

All the described ruthenium complexes proved to be poorly stable in aqueous media, where the most labile ligands, typically chloride ions, are easily and relatively rapidly replaced by hydroxide ions and/or water molecules, resulting in hydrolysis of the complexes [85,88,89,90,91,92,93,94,95], also leading to poly-oxo species formation [83,89,96,97]. Although it has been reported, at least for NAMI-A, that the presence of these oligomeric species does not really impair the overall anti-tumour activity [89,98], the premature formation of aquated species in the extracellular medium could deactivate, or activate too early, most of the administered drug, thus reducing the available active forms which ultimately interact with the biological targets (proteins or DNA) [3,99]. For this reason, the effective biomedical potential of these low molecular weight ruthenium complexes, administered as such, has been recently reconsidered. Taking into account that the ligand exchange process could also represent a potential mechanism of action of the Ru(III)-based drugs, or of activation in case they behave as prodrugs of the more reactive Ru(II)congeners, several efforts have been made to retard the ligand exchange processes in the extracellular environment so to ensure their occurrence once the drug has reached the cell.

Following the investigations carried out on KP1019 and NAMI-A [72], in a recent study the subcellular accumulation of AziRu was determined by inductively coupled plasma-mass spectrometry (ICP-MS) analysis performed on various biological samples taken from MCF-7 breast cancer cell cultures (culture medium, cellular pellet, cytosolic fraction, nuclear fraction and DNA samples) after 24 h of in vitro ruthenium treatment [100]. About 80% of the administered ruthenium was found in the culture medium, while only a very small amount (less than 10%) was detected in the nuclear fraction [100]. This result corroborated the hypothesis of a massive early drug deactivation in the extracellular environment and/or a poor ability to penetrate the cell membranes, reinforcing the need to protect the metal core in Ru-based drugs in order to obtain more efficient cell internalization.

Thus, in order to produce more effective Ru(III)-based anticancer agents, able to cross the biological barriers, our research group proposed a prodrug approach. In this design, the active unit, AziRu, has been incorporated into highly functionalized nucleolipid-based scaffolds [101] able to form, under physiological conditions, stable self-assembling aggregates efficiently protecting the metal complex from biological degradation and conveying it through the cell phospholipidic bilayers [77,79,102,103,104,105,106,107,108,109].

**Figure 2:**
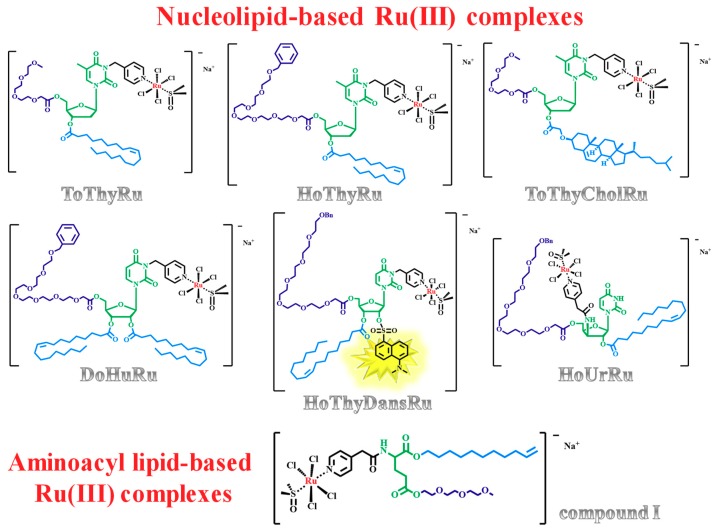
Chemical structures of the proposed nucleolipid and aminoacyl lipid-based Ru(III)-complexes. In the figures, ruthenium ions are indicated in red, the central scaffolds in green, while the hydrophilic and lipophilic chains in dark and light blue, respectively. In the HoThyDansRu molecular structure, the fluorescent dansyl group is highlighted in yellow (Bn = benzyl group).

In detail, the low molecular weight complex AziRu was decorated using ribo- and deoxyribonucleosides as starting building blocks, functionalized with suitable hydrophilic (oligoethylene glycol moieties) and lipophilic (oleoyl or cholesteroxyacetyl groups) chains, obtaining a mini-library of amphiphilic nanovectors to enhance the Ru(III) delivery in vivo. Within this approach, the nucleolipid-based Ru(III) complexes—named ToThyRu, HoThyRu, DoHuRu [77,106], ToThyCholRu [79,105], the fluorescently-labelled HoThyDansRu [100,106] and the second-generation HoUrRu [107] (Figure 2)—were successfully prepared and evaluated in their physico-chemical properties (for a recent review covering their design, synthesis and characterization, see Riccardi et al. [35]).

In order to further expand the chemical diversity of the available amphiphilic Ru(III) complexes, a central core alternative to the nucleolipidic one, i.e., the trifunctional α-amino acid glutamic acid, was explored to build the nanocarrier for the Ru(III) metal core [110]. The novel aminoacyl lipidic Ru(III) complex **I** (Figure 2) showed a slower hydrolysis kinetics compared to AziRu, demonstrating that also an aminoacyl lipid is a suitable scaffold to protect the Ru(III) complex from the aquation processes [110].

## 2. Ru(III)-Containing Formulations as Efficient Drug Delivery Systems

### 2.1. KP1019-Hosting Nanosystems

**Figure 3:**
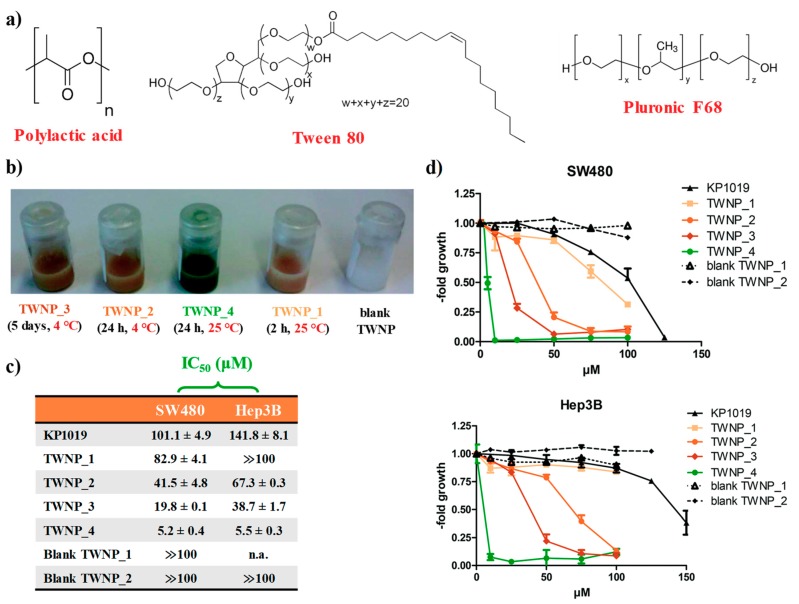
KP1019 incorporation into polylactic acid nanoparticles (PLA NPs): (**a**) chemical structures of polylactic acid and of the surfactant used in the formulation (poloxamer Pluronic F-68 and polysorbate Tween 80); (**b**) nanoparticle appearance after different temperature and period storage: TWNP_3 (5 d, 4 °C), TWNP_2 (24 h, 4 °C), TWNP_4 (24 h, 25 °C), TWNP_1 (2 h, 25 °C), blank TWNP, from left to right; (**c**) IC_50_ values (µM) of the different KP1019 nanoformulations prepared in comparison with naked KP1019. Blank TWNP_1 (1 month, 4 °C), Blank TWNP_2 (2 h, 25 °C); IC_50_ values are reported as mean ± SEM. (**d**) dose-response curves of colon carcinoma SW480 and the hepatoma Hep3B cancer cell lines with the indicated drugs as determined by MTT assay after 72 h treatment. Figures were adapted from [111] published by The Royal Society of Chemistry.

In order to overcome the poor stability observed for KP1019 in aqueous solutions, especially at physiological pH, Keppler et al. proposed an interesting approach [111]. KP1019 was entrapped in poly(lactic acid) nanoparticles (PLA NPs) in the presence of two different non-ionic surfactants: the poloxamer Pluronic F-68 and polysorbate Tween 80 (Figure 3a), in order to evaluate the contribution of the surfactant on the chemical stability and encapsulation properties of the final nanoaggregates [111]. These formulations, prepared by a single oil-in-water (o/w) emulsion and characterized by dynamic light scattering (DLS) and transmission electron microscopy (TEM) analyses, were essentially based on physical encapsulation, which exploits the affinity between the carrier and the drug to entrap the latter one in a suitable matrix.

The Ru-containing nanoaggregates based on Pluronic F-68 (indicated as PLNP) clearly showed a brown drug precipitation after ca. 15 h, suggesting that KP1019 diffused out of the PLNP and therefore this kind of nanoformulation was not further explored [111].

On the contrary, the use of Tween 80 allowed preventing drug precipitation with very high KP1019 loading efficiency, ranging from 92% to 95% with respect to the initial amount. When stored at 4 °C, the obtained formulations (indicated as TWNP) did not show settling of particles, indicating a substantial stability without agglomerate formation for about 1 month. In turn, these polysorbate-containing NPs, when left at r.t., showed a remarkable colour change from brown to deep green after ca. seven days, indicative of the reduction of the Ru(III) centre (Figure 3b) [111].

This hypothesis was then investigated by kinetic studies: ESI-MS experiments demonstrated the replacement of a chloride ligand by Tween 80, involving its moderately basic oxygen groups. This exchange reaction was also accompanied by reduction of the paramagnetic Ru(III) to the diamagnetic Ru(II) ion, as revealed by electron spin resonance (ESR) spectra, and explained by the well-known autoxidation ability of polysorbates [111].

The evaluation of the in vitro anticancer properties performed on colon carcinoma SW480 and hepatoma Hep3B cell lines indicated a modest increase of the antiproliferative activity for the stable NPs (stored at 4 °C), along with a markedly higher cytotoxic activity for the NPs left at r.t. (Figure 3c,d). Indeed, green NP solutions showed a 20- and 26-fold increased cytotoxic activity with respect to the naked KP1019 drug, respectively on SW480 and Hep3B cancer cells. Thus, surprisingly, longer storage period distinctly increased the activity of the KP1019-loaded TWNP particles [111].

For KP1019, the same research group also proposed a strategy alternative to the physical encapsulation, based on the chemical conjugation of the ruthenium complex with a selected polymer [112]. In particular, KP1019 was loaded into micelle-like carriers (MC-KP1019) formed by an ad hoc synthesized PEGylated polymer. Indeed, micelles proved to be highly biocompatible and efficient [113,114]. The obtained micelles were fully characterized by TEM and zeta potential measurements in order to determine their size range and surface charge. In addition, MC-KP1019 solutions (0.3 mg/mL KP1019) proved to be stable at 4 °C, with no precipitation for more than three months [112].

Then, the anticancer activity of MC-KP1019 was evaluated following 72 h drug incubation and compared with naked KP1019 on different human cancer cell lines, i.e., the colon carcinoma cell line HCT116 and its subline HCT116 (p53/ko) with a deleted p53 gene, the non-small cell lung carcinoma cell line SW1573 with its ABCC1- and LRP-overexpressing subline 2R120, and its ABCB1- and ABCC1-overexpressing subline 2R160, the epidermal carcinoma cell line KB-3-1 and its ABCB1-overexpressing subline KBC-1, the breast cancer cell model MCF-7 and its ABCB1-overexpressing subline MCF-7/adr, along with the human colon adenocarcinoma Lovo and its ABCB1- overexpressing subline Lovo/dox. In all cases, KP1019-containing micelles were found to be more active than naked KP1019, showing IC_50_ values between 1.6- and 22.7-fold lower compared to the free Ru compound (IC_50_ values ∼100 µM or above) [112].

Notably, in the SW1573 sublines 2R160 and 2R120, both being distinctly resistant to KP1019, the micellar formulation of KP1019 was able to overcome this resistance, showing very interesting cytotoxicity [112]. Moreover, the micelle-based nanosystems facilitated the cellular accumulation of KP1019, as determined by ICP-MS measurements. Concerning their mode of action, increased cell cycle arrest in G2/M phase (PI-staining), DNA damage (Comet assay) as well as enhanced levels of apoptotic cell death (caspase 7 and PARP cleavage) were found in HCT116 cells treated with the MC-KP1019 nanoformulations [112].

### 2.2. NAMI-A-Hosting Nanosystems

The micellization approach has been explored to enhance also the in vivo delivery of NAMI-A.

**Figure 4:**
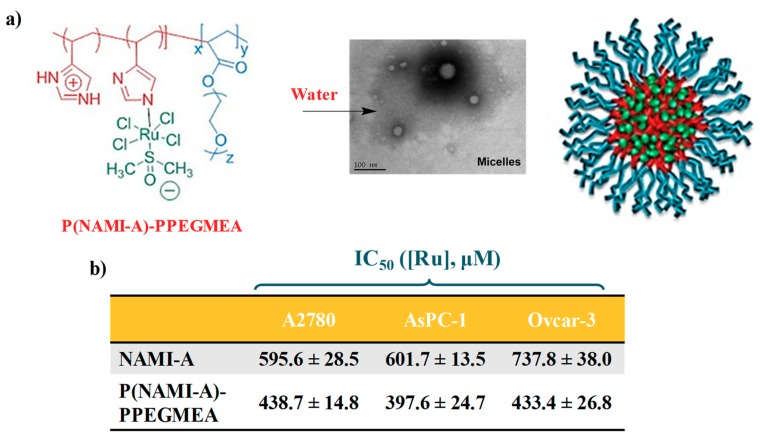
NAMI-A conjugation to polymeric micelles: (**a**) chemical structure of the amphiphilic block copolymer P(NAMI-A)-PPEGMEA and schematic representation of its micellization in water; (**b**) cytotoxicity and IC_50_ values (µM, expressed in terms of ruthenium concentration) of NAMI-A and P(NAMI-A)-PPEGMEA against ovarian A2780 and Ovcar-3 and pancreatic AsPC-1 cancer cell lines. IC_50_ values are reported as mean ± SEM. Figures were adapted with permission from [115] Copyright 2014 from American Chemical Society.

Stenzel and coworkers reported the polymerization of 4-vinil imidazole (VIm) using a trithiocarbonate RAFT agent, i.e., 2-[(dodecylthio-carbonothioyl)thio]-2-methylpropanoic acid, and the subsequent chain extension with poly(ethylene glycol)methyl ether acrylate (PPEGMEA) to obtain a biocompatible amphiphilic block copolymer capable to self-assemble into polymeric micelles and incorporate NAMI-A (indicated as P(NAMI-A)-PPEGMEA, Figure 4a) [115].

The polymerization reaction was followed by SEC analysis monitoring the retention time changes due to the increased polymer size over time and the resulting micelles were then characterized by DLS and TEM experiments (Figure 4a) [115].

When tested on ovarian (A2780 and Ovcar-3) and highly aggressive pancreatic AsPC-1 cancer cell lines, a 1.5-times increase in cytotoxicity was found in all the tested cell lines for the polymeric NAMI-A-containing micelles compared to the naked ruthenium complex (Figure 4b) [115].

Notably, polymeric micelles significantly improved also the NAMI-A antimetastatic potential, with increased inhibitory effects on both the migration and invasion of human breast cancer cells [115]. In particular, the influence of the polymeric P(NAMI-A)-PPEGMEA micelles on the migration processes of three cell lines (invasive cancerous MDA-MB-231, noninvasive cancerous MCF-7 cancerous noncancerous CHO) was evaluated when a chemical (chemotaxis) and a contact (haptotaxis) stimulus was applied to promote cell movements. Both the chemotactic and haptotactic migration of breast cancer cells were inhibited in a massive way with respect to the nontumourigenic CHO cells, with statistically significant effects observed for the invasive MDA-MB-231 cell line [115].

### 2.3. AziRu-Hosting Nanosystems

**Figure 5:**
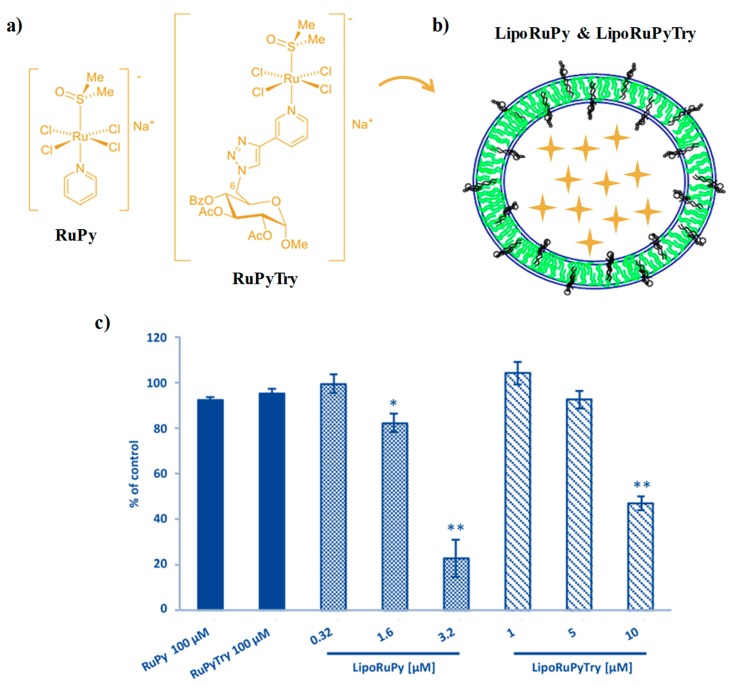
Pyridine-containing Ru(III) complexes inserted in liposome systems: (**a**) chemical structure of the proposed RuPy and RuPyTry compounds; (**b**) schematic representation of liposomal nanoaggregates containing the pyridine-based Ru(III) complexes; (**c**) dose-response histograms reporting the cytotoxic effects observed for the naked Ru(III) compounds and their liposomal formulations in PC-3 cancer cells after 48 h treatment. Figures are adapted from [116].

Recently, Tesauro and colleagues proposed a variant of the well-known AziRu (indicated in this work as RuPy by the authors) containing a fully protected sugar moiety as decoration for the ruthenium-coordinating pyridine moiety (named RuPyTry, Figure 5a) aiming at increasing the Ru(III)complex cell internalization [116]. This novel complex, fully characterized by ESI-MS, IR and ^1^H NMR spectroscopy, showed a faster hydrolysis in aqueous solution with respect to its parent compound, with formation of the respective monoaqua complex, probably favoured by the hydrogen-bonding properties of the nitrogen atoms in the triazole moiety facilitating the access of water molecules to the metal [116].

Then, Ru-containing liposome formulations were prepared mixing phosphatidylcholine (PC)/cholesterol (Chol)/DSPEPeg2000 in 57/38/5 molar ratio (Figure 5b). The obtained liposomes, with ca. 100 nm size, exhibited very slow ruthenium release kinetics, with only 4% of the complex released in serum over 72 h monitoring [116].

These Ru(III) complexes as such, as well as their liposomal nanoaggregates, LipoRuPy and LipoRuPyTry, were evaluated for their anticancer activity analysing the inhibition of cell proliferation on human prostate cancer PC-3 cells and human dermal fibroblasts (NHDF).

The naked Ru(III) complexes did not show significant cytotoxic effects on cancer PC-3 cells even when tested at 100 μM concentration (Figure 5c); a slight decrease in cell viability was found only at concentrations >500 μM. Conversely, the liposomal formulations exhibited relevant cytotoxicity under the same conditions. The observed reduction in cell viability was found to be concentration-dependent and more marked for the RuPy-containing liposomes (LipoRuPy, Figure 5c). In turn, when tested on normal NHDF cells, these formulations did not affect cell viability even at 100 μM concentration, demonstrating their selectivity and promising bioactivity [116].

### 2.4. Mononuclear and Dinuclear Ru(III)-Dithiocarbamato Complexes Encapsulated in Nanosized Carriers

**Figure 6:**
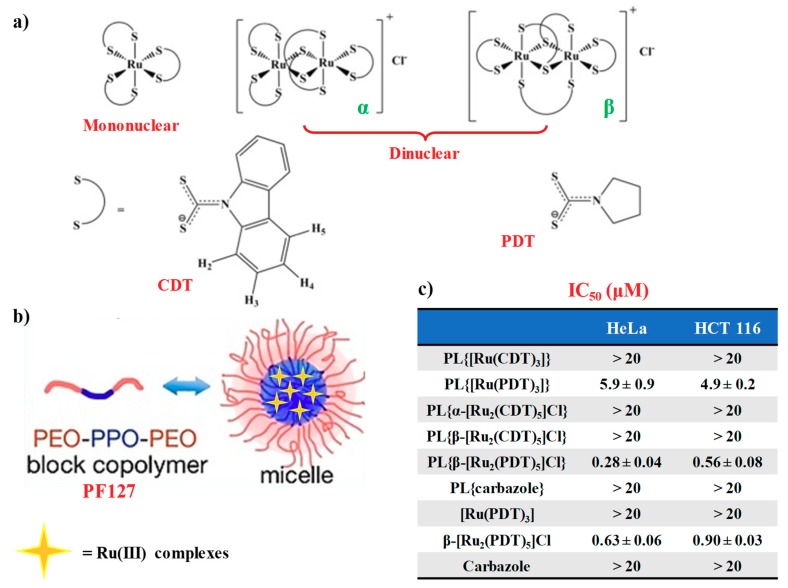
(**a**) Chemical structures of mononuclear and dinuclear Ru(III)-dithiocarbamato complexes and of the investigated dithiocarbamato ligands: pyrrolidyl dithiocarbamate (PDT) and carbazolyl dithiocarbamate (CDT); (**b**) schematic representation of the Pluronic^®^ F127 block copolymer and micelle formation with Ru(III) complex internalization; (**c**) IC_50_ values (μM) evaluated after 72 h treatment with free Ru(III)-dithiocarbamato complexes and their micelles formulations (indicated as PL{compound}). IC_50_ values are reported as mean ± SEM. Figures are adapted from [117].

Fregona et al. [117] proposed novel mono- and dinuclear Ru(III)-complexes with aromatic and non-aromatic dithiocarbamates as ligands: in particular, carbazolyldithiocarbamato derivatives (CDT) and pyrrolidinedithiocarbamate (PDT) analogues were prepared (Figure 6a). These compounds were fully characterized by physico-chemical techniques such as elemental analysis, ESI-MS spectrometry, ^1^H NMR UV–VIS and FT-IR spectroscopic studies [117].

In order to address the solubility issues typically associated with ruthenium drugs under physiological conditions, both CDT and PDT Ru(III)-based derivatives were encapsulated in water-soluble micellar carriers by using the biocompatible copolymer Pluronic^®^ F127 [117].

Such block copolymer is a non-ionic surfactant consisting of hydrophilic poly(ethylene oxide) (PEO) and hydrophobic poly(propylene oxide) (PPO), arranged in A-B-A tri-block structure which comprise a hydrophobic PPO region covered by a hydrophilic shell, made up of PEO chains [118].

The incorporation of the selected Ru(III)-based compounds during the micellization process was driven by the hydrophobic interactions established between the compounds and the PPO domain of the selected copolymer, hence preventing unwanted release of the loaded compounds (Figure 6b for the schematic representation of the micelle formation) [117]. As verified by UV–VIS analysis in saline solution (NaCl 0.9% *w*/*v*), the prepared Ru(III)-incorporating micellar nanocarriers did not show significant changes in the UV spectra over time, at least over 72 h monitoring [117].

Both naked CDT- or PDT-based ruthenium compounds as well as their nanoformulations were then tested in preliminary bioactivity assays on two human tumour epithelial cell lines, i.e., HeLa (cervix adenocarcinoma) and HCT 116 (colon carcinoma) over 72 h treatment, using cisplatin as the control [117].

Interestingly, the β form of the PDT dinuclear complex ([Ru_2_(PDT)_5_]Cl) evidenced a ca. two-fold higher activity when loaded into Pluronic^®^ F-127 micelles compared to its naked form. This occurred also for the mononuclear PDT complex, showing a three-fold increased anticancer activity (Figure 6c) [117]. Notably, all the PDT-based compounds proved to be more efficient against cervical HeLa than on colon carcinoma HCT116 cancer cell lines, as also found for the Ru(III)-containing nanosystems [109]. Conversely, both mononuclear and dinuclear Ru(III)-CDT complexes encapsulated in Pluronic^®^ F127 micelles were not active on the tested cell lines [117].

### 2.5. Liposome-Based Systems Containing Nucleolipid or Aminoacyl Lipid-Based Ru(III) Complexes

**Figure 7:**
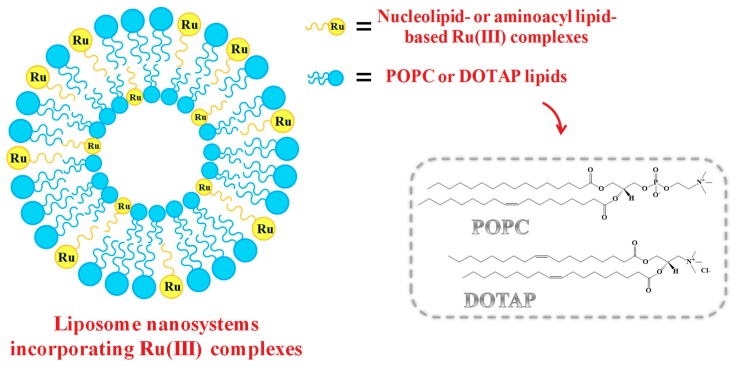
Schematic representation of liposomal POPC- or DOTAP-based nanoaggregates containing nucleolipid- or aminoacyl lipid-based Ru(III) complexes.

As in the case of low molecular weight Ru(III) compounds, also the nucleolipid- or aminoacyl lipid-based complexes incorporating AziRu, here described in paragraph 1.3, when dissolved in aqueous solutions, showed tendency to hydrolyse over time, even if sensibly reduced compared to naked AziRu [77,105,110]. In order to further increase their stability and obtain liposome-based delivery systems incorporating specific amounts of the selected Ru(III) complex, our group studied the co-aggregation of both nucleolipid- and aminoacyl lipid-based Ru(III) complexes with either the zwitterionic phospholipid POPC (1-palmitoyl-2-oleoyl-sn-glycero-3-phosphocholine) or the cationic lipid DOTAP (1,2-dioleoyl-3-trimethylammoniumpropane). A schematic representation of the obtained liposome-based nanosystems is reported in Figure 7.

Liposomes, discovered in the 1960s, consist in phospholipid-based nanovesicles having a morphology very similar to cellular membranes, with an aqueous core surrounded by lipid bilayers [119,120,121]. Thanks to their peculiar structure, liposomes are considered ideal nanosystems to encapsulate both hydrophilic and lipophilic drugs, respectively in the aqueous core and within the lipid bilayer [122,123,124]. To date, liposomes also represent the most successful drug delivery systems [125], with several formulations approved by FDA for anticancer therapy and also available in clinical use [119,126,127,128,129], such as lipoplatin, a liposomal cisplatin formulation approved by FDA for pancreatic and lung cancer treatment [130].

Thus, POPC and DOTAP lipids were chosen as Ru(III) complexes stabilizing agents on the basis of their proved biocompatibility [77,105,106,131,132]. In particular, POPC liposomes are efficiently used in a broad range of biotechnological applications [133], while liposomes formed by the cationic lipid DOTAP showed noteworthy transfection efficiency both in vitro and in vivo [131], probably due to the favourable interactions with the negatively charged membranes.

The use of phospholipids—forming ordered bilayers in which the Ru complexes can be easily lodged—allowed obtaining Ru(III)-containing formulations stable for months in physiological media, in which the incorporated metal amount can be finely modulated.

On the other hand, the selected lipids allowed exploiting different interactions for the Ru-complex embedding (mostly hydrophobic for POPC, and both hydrophobic and electrostatic for DOTAP), thus providing a basis to evaluate a structure-activity rationale.

Generally, the Ru(III)-containing POPC formulations were prepared mixing the Ru complex and the lipid in 15:85 molar ratio [77,105,107]. In turn, liposomes based on cationic DOTAP lipid were able to incorporate a higher amount of the nucleolipid- or aminoacyl lipid-based Ru complex, providing stable DOTAP co-aggregates with up to 30% in moles of ToThyCholRu [79] or the aminoacyl lipid compound **I** [110], and 50% in moles of ToThyRu, HoThyRu, DoHuRu or HoUrRu [106,107]. Both Ru(III)-containing liposomes showed effectively retarded ligand exchange processes in physiological solutions [105,110].

On these Ru(III)-containing liposomes detailed preclinical studies were then carried out in order to assess their anticancer activity and disclose their mechanism of action, so to evaluate their potential in cancer treatments.

## 3. Antiproliferative Effects of Liposome-Based Systems Containing Nucleolipid or Aminoacyl Lipid-Based Ru(III) Complexes: Insight into Their Mode of Action

### 3.1. In Vitro Bioactivity

In order to verify the antiproliferative efficacy of the nucleolipid- or aminoacyl lipid-based Ru(III) complexes inserted in POPC- and DOTAP-based formulations, detailed investigations on their in vitro bioactivity were performed in comparison with naked AziRu [77,79,100,105,106,107,110,134]. A selected panel of human and non-human cancer cell lines was chosen, particularly focusing on cells of different histopathological origin and widely used in anticancer research due to their replicative potential and malignancy, such as MCF-7 (human breast adenocarcinoma cell line), WiDr (human epithelial colorectal adenocarcinoma cell line), HeLa (human cervical cancer cells) and C6 (tumour rat glioma cells) [134,135,136]. In parallel experiments, some of these formulations were also tested on normal cell lines, such as L6 (rat muscle cells) and HaCaT (human keratinocytes cells), representing useful models to assess possible biological effect on healthy cells [137]. The experimental procedures here adopted involved the estimation of the anticancer activity by means of a ‘‘cell survival index’’, arising from the combination of a mitochondrial functional assay in vitro for the evaluation of the metabolic activity with an automated cell count. From the resulting concentration/effect curves, IC_50_ values were determined (Table 1).

In line with our expectations, liposomes exclusively composed by POPC or DOTAP did not interfere with cell viability and proliferation [77,105,106]. On the contrary, all the tested Ru(III)-containing POPC- and DOTAP-based formulations inhibited cancer cell proliferation at least one order of magnitude more effectively than naked AziRu, with IC_50_ values in the low μM range, indicative of a significant activity in reducing cancer cells growth (Table 1) [35,77,79,100,105,106,107,110,134].

Thus, the insertion of AziRu into highly functionalized scaffolds (nucleolipid or aminoacyl lipid structures), along with the subsequent liposome encapsulation, allowed us converting a very weakly cytotoxic compound into effective antiproliferative agents, with IC_50_ values from ∼4 to ∼50 times lower than the parent metal complex [35,77,79,100,105,106,107,110,134].

The higher anticancer activity observed for the tested formulations with respect to the naked AziRu suggested an enhanced cellular uptake efficiency for the Ru(III)-incorporating liposomes due to nanovectorization [35,77,79,100,105,106,107,110,134]. Interestingly, when tested on healthy cell lines, as human HaCaT keratinocytes and rat L6 muscle cells, the Ru(III)-containing liposomal formulations did not show toxicity even at very high concentrations, demonstrating a selective cytotoxicity against highly proliferative malignant cells (Table 1).

These in vitro outcomes placed these Ru(III)-containing liposomal formulations among the most promising Ru-based anticancer agents currently described in the literature [35,77,79,100,105,106,107,110,134], with only few reported cases showing similar in vitro antiproliferative activity [80,111,116,117,138,139,140,141].

Furthermore, in most investigated cancer cells, the IC_50_ values for the DOTAP formulations were significantly lower than those calculated for the same Ru(III) complex lodged in POPC liposomes [35,79,100,106,107,134]. This indicates that, under identical experimental conditions, the cationic DOTAP liposomes greatly enhanced the anticancer activity of the nucleolipidic Ru(III) complexes, likely due to the positive net superficial charge of the nanocarriers which can promote the interaction with cell membranes and, consequently, the drug internalization.

Interestingly, the differences among the various nucleolipidic Ru(III) complexes incorporated in the liposomal formulations were not very significant, consistently with the rationale that the metal centre is the effectively bioactive species and the different nucleolipids simply represent useful variants of the carrier molecules [35].

From these bioscreens, MCF-7 breast cancer cells proved to be the most sensitive to ruthenium treatments in vitro: for this reason, the evaluation of the cellular response to the exposure to some of the developed Ru(III)-containing formulations was extended to other in vitro models of human breast cancer cells (BCC), worldwide the most common invasive cancers in women [142,143,144]. In particular, ToThyRu and DoHuRu-containing liposomes (both POPC and DOTAP formulations) [134] as well as HoThyRu/DOTAP formulations [100] were further investigated focusing on well-established breast cancer cell lines: in addition to the epithelial-like breast adenocarcinoma MCF-7 cells, MDA-MB-231, MDA-MB-436, MDA-MB-468 and CG5 cells were inserted into a panel of human breast cancer models for preclinical evaluations (Table 2). The endocrine-responsive (ER) breast adenocarcinoma MCF-7 and the triple-negative breast adenocarcinoma (TNBC) MDA-MB-231 cell models account for the great majority of investigations on breast cancer cells and are considered the most reliable in vitro models of breast cancer together with their variants CG5, MDA-MB-436 and MDA-MB-468, respectively [145,146,147].

According to the reported IC_50_ values (Table 2), all the liposome formulations were effective in reducing cell growth and proliferation, proving the high sensitivity of BCC to the action of Ru(III)-based nanoaggregates, mainly of DOTAP-based formulations [134]. Notably, HoThyRu/DOTAP was basically inactive when tested on MCF-10A cells (IC_50_ higher than 100 µM considering the effective ruthenium concentration), used as a reliable model for normal human mammary epithelial cells. Taken together, these outcomes proved a significant selectivity of these Ru(III)-hosting nanosystems towards BCC lines, without significant biological effects on healthy counterparts of mammary gland as epithelial cells [100,134].

### 3.2. Cellular Uptake Studies on Ru(III)-Containing Liposomes by Fluorescence Microscopy

In vitro preliminary investigations demonstrated that all the Ru(III)-containing liposomes were endowed with significant antiproliferative activity toward cancer cells of different histological origin, with particular efficacy on in vitro models of breast cancer cells.

Since one possible mechanism of action of the ruthenium-based anticancer drugs was suggested to be the nuclear DNA binding [148,149], the cellular uptake and nuclear entry ability were investigated, being a key element to induce inhibition of cancer cell proliferation [72].

Thus, time-course fluorescence microscopy experiments were performed to evaluate the Ru(III)-containing liposome uptake and their subcellular localization in carcinoma cells at different incubation times. In particular, ToThyRu/POPC formulations as well as DOTAP-based liposomes containing either DoHuRu or ToThyCholRu were evaluated in parallel experiments as representatives respectively of POPC and DOTAP-based nanoaggregates [77,79,106]. In the case of the cellular uptake evaluation of ToThyRu/POPC and DoHuRu/DOTAP nanoformulations, breast MCF-7 cancer cells were selected. In turn, the in vitro uptake of ToThyCholRu/DOTAP nanosystems was evaluated on both MCF-7 and colorectal WiDr cancer cell lines, in order to verify possible differences toward cancer cells of different histological origin.

In these experiments, the liposomes were loaded also with the fluorescent rhodamine-B (Rhod) dye, allowing to reveal their fusion with the membranes and cellular uptake; the blue fluorescent stain DAPI was used to label DNA, in order to detect the position of the nuclei; finally, merged images, arising from overlapped fluorophore emissions of DAPI and Rhod from the same cell monolayers, gave a clear and unambiguous indication on the overall cellular uptake process and its kinetics.

For all the investigated formulations, independently from the analysed cancer cell line, a marked fluorescence was detected into cells already after 1 h incubation. After typically 3 and 6 h, fluorescence localized widespread in the nuclei, cytoplasm and close to the membranes, indicating that the liposomes were significantly taken up by cancer cells [77,79,106]. Few differences in the cell uptake were found on varying the lipid used in the formulation: indeed, in the case of DOTAP nanoaggregates hosting DoHuRu or ToThyCholRu complexes, a massive cell uptake was already observed after 30 min incubation [77,79,106]. Therefore, in all cases these formulations showed a high propensity to cross the cell membranes along the uptake process in the tested cancer cell lines [77,79,106].

**Figure 8:**
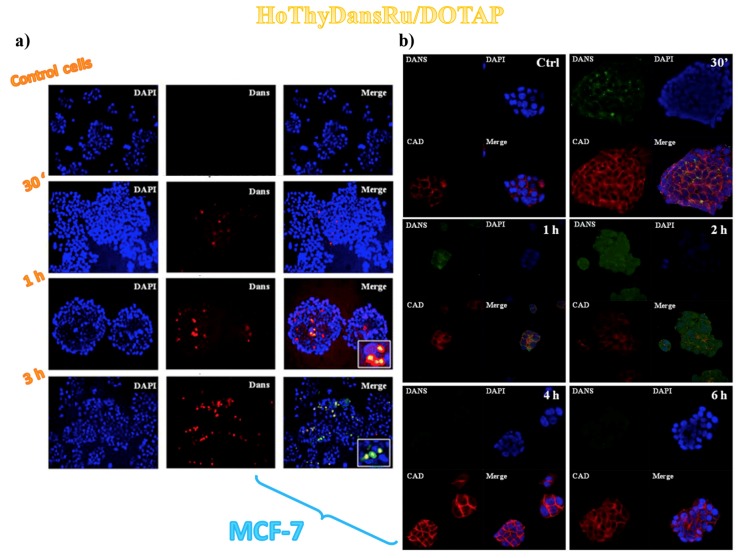
Human MCF-7 breast adenocarcinoma cells incubated with 100 µM HoThyDansRu/DOTAP liposome formulations and followed at different times: (**a**) fluorescent photomicrographs of cell monolayers showing the cellular localization of the fluorescent HoThyDansRu/DOTAP liposomes; (**b**) confocal microscopy bioimaging showing the cellular uptake of the fluorescent HoThyDansRu/DOTAP nanosystem. DAPI was used as a nuclear stain (shown in blue). Dansyl-dependent fluorescence (Dans) of HoThyDansRu/DOTAP liposomes is shown in red (**a**) or as green dots (**b**). E-cadherine-associated fluorescence (CAD), defining cell shape, is shown in red (**b**). In merged images (Merge), the two fluorescent emission patterns are overlapped. Insets in panel **a** represent higher magnifications of merged images showing cellular location of fluorescent dansylated ruthenium complexes. Figures were adapted with permission from [106] Copyright (2013) American Chemical Society (**a**) and from [100] (**b**).

Aiming at a deeper insight into the cell internalization process and metabolic fate of the liposomal formulations, additional in vitro fluorescence experiments were performed on MCF-7 cancer cells treated with an ad hoc prepared dansyl-labelled ruthenium complex named HoThyDansRu (Figure 2), co-aggregated with DOTAP analogously to the other described nucleolipid-based complexes. Monitoring the dansyl fluorescence, the fate of the active ruthenium complex could be directly assessed, examining its localization after cells treatment. Cationic liposomes hosting the fluorescently labelled HoThyDansRu rapidly localized within the cells, showing relevant cytoplasm dansyl-dependent fluorescence emission already after 30 min incubation and increasing accumulation over time, reaching the maximum fluorescence emission after 2 h (Figure 8a) [106]. A marked tendency to accumulate in proximity of the nuclei was detected (Figure 8a), suggesting possible interaction of the active metal also with nucleic acids [106].

Overall, analysis of the dansyl-dependent fluorescence emission over time indicated that the HoThyDansRu/DOTAP nanoaggregates first localized within the cell membranes, then entered the cytoplasm, spreading to the whole cell, including perinuclear and nuclear compartments (Figure 8b) [100].

The fluorescence detection suggested an intracellular release of the ruthenium derivative from the liposomes: at longer incubation times (i.e., 4 and 6 h), a significant decrease in the dansyl-dependent fluorescence signal intensity occurred (Figure 8b), supporting the hypothesis that the liposome released the active ruthenium complex inside the cell [100]. In fact, it was demonstrated that the fluorescence intensity of the dansyl probe is largely influenced by the external environment: the fluorescence of dansyl group is marked in hydrophobic environments, while poor fluorescence emission is detected when the probe is exposed to water [150,151,152]. Accordingly, the marked fluorescence intensity of HoThyDansRu in the first monitoring indicated that the dansyl dye was inserted in the apolar DOTAP lipid bilayer; its progressively less and less intense fluorescence (Figure 8b) suggested that the nucleolipid Ru-complex was released in the intracellular media [100].

All these findings were thus corroborated in MCF-7 cells by fluorescence experiments using confocal microscopy coupled to Red E-cadherine-associated fluorophore, a 120-kDa transmembrane glycoprotein able to highlight adherens junctions and cell-cell contacts in epithelial clusters [100].

Overall, these data highlighted the crucial role played by the cellular uptake process in determining the anticancer efficacy of Ru(III)-based drugs, showing both POPC and DOTAP-based liposomes as very efficient nanocarriers for the stabilization of Ru complexes in aqueous media and their effective transport in cells. Particularly relevant was their clear detection in the nucleus, differently from other known Ru(III)complexes, able to accumulate in the cytoplasm but not in the nuclear region [153]. Notably, for these systems cationic DOTAP nanoaggregates proved to be more effective as Ru(III)-based drugs carriers, showing faster uptake kinetics [77,79,100,106].

### 3.3. Sub-Cellular Accumulation of the Ru(III) Complexes

Ruthenium intracellular amount was determined by ICP-MS analysis performed on subcellular fractions (culture medium, cellular pellet, cytosolic fraction, nuclear fraction and DNA sample) of MCF-7 cells, after 24 h in vitro treatment with HoThyRu/DOTAP nanoaggregates [100]. AziRu was also included as suitable control, demonstrating the vital importance of nanoformulations for an efficient ruthenium cellular uptake and internalization [100]. In fact, after 24 h exposure, about 80% of the administered AziRu was found in the culture medium, while 85% of the administered HoThyRu/DOTAP liposomes amount was revealed at cellular level.

Furthermore, analysis of the isolated subcellular fractions indicated for the HoThyRu/DOTAP formulations a broad ruthenium distribution among the intracellular compartments, above all in the nucleus. Almost 50% of the ruthenium administered as liposomal nanosystem was found bound to nuclear DNA vs. <10% of the metal from the naked AziRu detected in the nuclear fraction bound to DNA [100].

### 3.4. Cell Morphological Changes Induced by in Vitro Treatment with Ru(III)-Containing Liposomes

To further support the relationship between cell viability and ruthenium-induced cytotoxicity, subconfluent cultures of the selected cancer cell lines throughout the in vitro experiments were also examined by phase-contrast light microscopy to monitor the dynamic morphological changes occurring during cell death. Thus, confluent MCF-7, WiDr and C6 cancer cells were evaluated after incubation with ToThyRu/POPC liposomes and MCF-7, WiDr and HeLa cells were examined after in vitro treatments with ToThyCholRu/DOTAP formulations, as representative example of POPC and DOTAP-based nanoaggregates, respectively.

**Figure 9:**
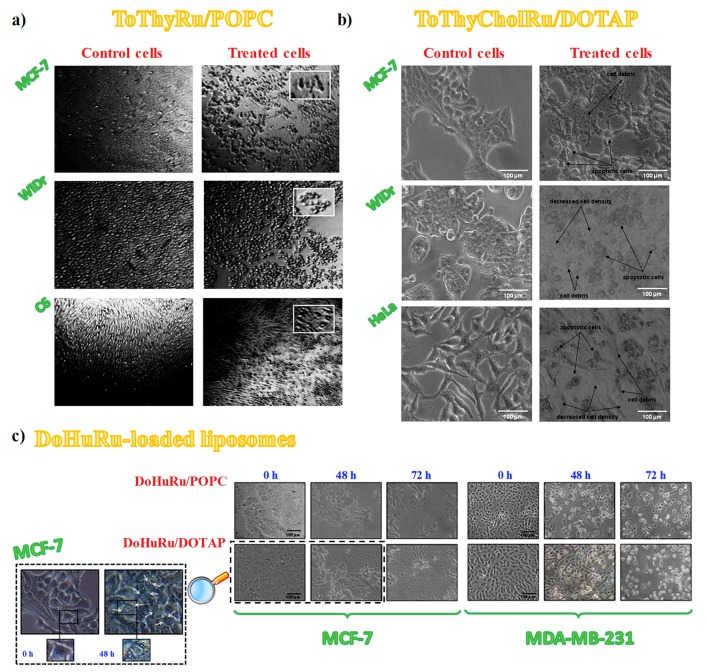
Representative photomicrographs by phase-contrast light microscopy showing the cellular morphological changes and cytotoxic effects on cellular monolayers: untreated (left column in panels **a** and **b**) or treated for 48 h with ToThyRu/POPC (**a**) or ToThyCholRu/DOTAP (**b**) formulations. For ToThyRu/POPC nanosystems, MCF-7, WiDr and C6 cancer cell lines were examined (**a**); in turn, for ToThyCholRu/DOTAP nanocarriers, MCF-7, WiDr and HeLa cancer cell lines were analysed (**b**). Insets in (a) represent higher magnifications of injured cells following incubations with ToThyRu/POPC liposomes. (**c**) MCF-7 and MDA-MB-231 breast cancer cells treated for 48 and 72 h with ruthenium IC_50_ concentrations of DoHuRu-containing liposomes. The inset in (c) represents higher magnifications at 0 (left panel) and 48 h (right panel) DoHuRu/DOTAP treated MCF-7 cells, showing the formation of autophagic vacuoles detectable in cell cytoplasm. Figures are adapted from [77] (**a**), [79] (**b**) and [134] (**c**).

In all cases, after 48 h in vitro treatments of the selected cancer cell lines, morphological modifications of the cell monolayers were clearly evident [77,79], with distinctive hallmarks of apoptosis (Figure 9a,b) [154]. Indeed, besides losing their normal morphology, apoptotic features—as membrane blebs and cell shrinkage [154]—appeared in the treated cells together with a significant increase of the number of rounded-up cells after exposure to the Ru(III)-containing liposomes [77,79].

In the case of cytomorphological modifications induced by the treatment with the aminoacyl lipid Ru(III) compound **I** hosted in DOTAP liposomes, not only malignant cells such as MCF-7, HeLa and C6 but also normal cell lines as 3T3-L1 (mouse embryo fibroblast) and HaCaT (human keratinocytes) were analysed by phase-contrast light microscopy in the absence and presence of the drug. Notably, while the cell distribution and cytomorphology were substantially unaffected on the healthy cells after 48 h of in vitro treatment, under the same conditions the cell monolayers integrity was strongly perturbed, with distinctive hallmarks of apoptotic cell death, such as membrane blebs, cell shrinkage, formation of balloon-like structures and cell contraction [154], in the case of the cancer cells [110].

More recently, cellular cytomorphological changes induced by Ru(III)-containing liposome administration were specifically investigated in two different breast adenocarcinoma cell lines, i.e., MCF-7 and MDA-MB-231 cells [146,147]. Indeed, in the presence of DoHuRu/POPC and DoHuRu/DOTAP formulations at 48 or 72 h [134], photomicrographs indicated the characteristic cell shrinkage as well as loss of cell-cell contacts, diagnostic of apoptosis (Figure 9c) [154].

Therefore, the overall morphological analysis outcomes provided strong evidence of an apoptosis-inducing activity, in line with several in vitro studies supporting apoptotic events to explain the anticancer properties of different ruthenium derivatives, both in their +2 and +3 oxidation states [47,78,112,140,141,153,155,156,157,158,159,160,161]. However, also other different molecular pathways can be involved in the cell death process [153]. In fact, in the case of MCF-7 cells treated with the DoHuRu/DOTAP liposomes at IC_50_ conc., in addition to apoptotic hallmarks, also autophagic vacuoles were clearly detectable, suggesting morphological changes associated with autophagy activation (Figure 9c, inset) [134].

### 3.5. Insight into the Ru(III)-Containing Liposomes Mode of Action: Identification of Molecular Cell Death Pathways

#### 3.5.1. Pro-Apoptotic Effects in Breast Cancer Cells Evaluated by FACS Analysis 

The observed changes both in the cell morphology and in the monolayers appearance suggested that Ru(III)-containing liposomal formulations might exert their antiproliferative effects in BCC by activation of specific cell death pathways, such as apoptosis and/or autophagy mechanisms [77,79,110,134].

To investigate the apoptosis induction in MCF-7 and MDA-MB-231 cancer cells, both DoHuRu-hosting POPC and DOTAP formulations were analysed by fluorescence-activated cell sorting (FACS) analysis by using Annexin V-FITC (fluorescein isothiocyanate) along with the propidium iodide (PI) dye, a very sensitive method to differentiate apoptotic (Annexin V-FITC positive, PI negative) from necrotic (Annexin V-FITC positive, PI positive) cells [162].

**Figure 10:**
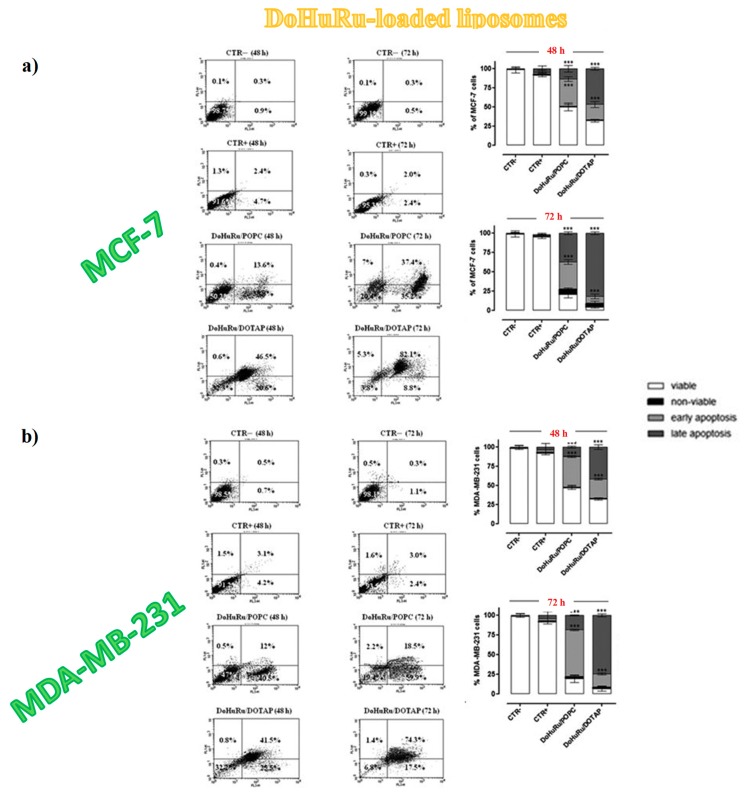
Induction of apoptosis in MCF-7 (**a**) and MDA-MB-231(**b**) breast cancer cells, evaluated by FACS analysis (right on each panel), after cell labelling with propidium iodide (PI) and FITC-Annexin V. Cells were both unlabelled and untreated (CTR −), labelled and not treated (CTR +), treated with IC_50_ doses of DoHuRu-incorporating POPC or DOTAP liposomes for 48 and 72 h, as indicated. The quantitative analysis of viable, non-viable (necrotic), early and late apoptotic cells after drug exposure is represented (bar graphs on the right of each panel). Viable cells are negative for both PI and FITC-Annexin V binding; non-viable, necrotic cells are negative for FITC-Annexin V binding and positive for PI uptake; cells in early apoptosis are FITC-Annexin V positive and PI negative; cells in late apoptosis are positive for both FITC-Annexin V binding and PI uptake. Figures were adapted from [134].

Both DoHuRu-incorporating lipid formulations induced exceptional pro-apoptotic effects, more marked for the DOTAP-based nanoaggregates, without observing significant increased necrosis (Figure 10) [134]. In particular, for MCF-7 cells, after 48 h of treatment at IC_50_ conc. of the tested formulations, 46% and 36% of total cell population, respectively, for DoHuRu/DOTAP and DoHuRu/POPC formulations, were found in early stages of apoptosis (Annexin V-FITC positive and PI negative). Additional 24 h treatment resulted in 82% and 37% of cell population, respectively, for DoHuRu/DOTAP and DoHuRu/POPC formulations, in late apoptosis phase (positive for both Annexin V-FITC binding and for PI uptake). Under the same conditions, similar trend and percentage of cell population in the different apoptotic stages were found also for MDA-MB-231 cells, once again with stronger pro-apoptotic effects for DOTAP- than POPC-based formulations (Figure 10) [134]. Indeed, these results further corroborated the increased in vitro efficacy—especially as a trend over time—of the cationic DOTAP nanocarriers with respect to the zwitterionic POPC liposomes hosting the same nucleolipidic Ru(III)-complex, likely due to different cellular uptake kinetics.

#### 3.5.2. DNA Fragmentation Assay

It is generally accepted that apoptosis activation and subsequent DNA cleavage represent the main cytotoxic mode of action of metal-based antiproliferative drugs [4,141]. In addition to shrinkage and fragmentation of cells and nuclei, the apoptotic processes are also accompanied by a marked degradation of the chromosomal DNA in the nucleosomal area. Late events of apoptosis typically lead to DNA fragmentation, resulting in a “ladder” formation easily detectable by agarose gel electrophoresis [163]. Thus DNA cleavage extent in cultured cells is directly correlated with the amount of apoptotic cells and this kind of investigation has been already explored for other Ru(III) complexes after in vitro treatment of MCF-breast cancer cells [112,141,153].

**Figure 11:**
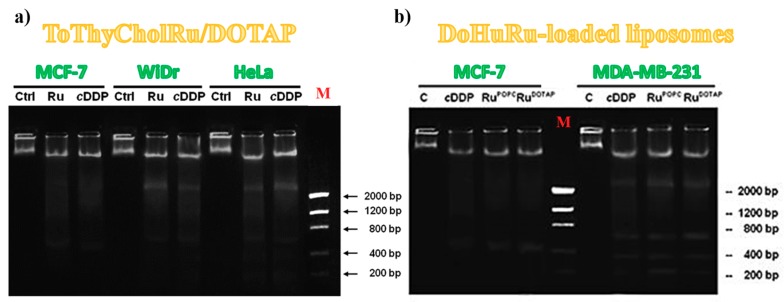
1.5% agarose gels representing the DNA fragmentation assay on MCF-7, WiDr and HeLa cells, treated or not (Ctrl) for 48 h with IC_50_ doses of ToThyCholRu–DOTAP liposomes (indicated as Ru) (**a**) and MCF-7 and MDA-MB-321 cells treated or not (C) with IC_50_ concentrations of DoHuRu/POPC (Ru^POPC^) and DoHuRu/DOTAP (Ru^DOTAP^) for 48 h (**b**). In both experiments, the selected cell lines were also treated with IC_50_ conc. of cisplatin (cDDP) used as a control. Lane M corresponds to the molecular weight markers. Figures are adapted from [79] (**a**) and [134] (**b**).

To further sustain the evidence that Ru(III)-containing formulations were able to trigger apoptosis in cancer cell lines, DNA degradation on genomic DNA samples obtained from treated cells was also investigated. In particular, ToThyCholRu/DOTAP liposomes were evaluated for their DNA fragmentation activity on MCF-7, WiDr and HeLa cancer cells (Figure 11a) [79]; while DoHuRu-hosting lipid formulations were specifically evaluated on both MCF-7 and MDA-MB-231 breast cancer cells (Figure 11b) [134].

In all cases, the DNA extracted from cells following 48 h of incubation with IC_50_ doses of Ru(III)-containing formulations strongly indicated fragmentation, accompanied by typical internucleosomal DNA laddering. The DNA cleavage extent was very close to that induced by cisplatin, used as positive control [163], while no relevant DNA fragmentation was found in untreated cancer cells (Figure 11) [79,134].

However, in all the tested formulations, the nuclear fragmentation pattern observed for MCF-7 cancer cells was not typical. This was explained considering that this cell line is particularly resistant to chemotherapeutics due to deletion in the CASP-3 gene, leading to an inherited caspase-3 deficiency expression [164]. Caspase-3 proteins are commonly activated by different death signals and are therefore responsible of cleavage of important cellular proteins and ultimately of apoptotic DNA damage [165]. In this case, MCF-7 cells undergo cell death triggered by apoptotic stimuli in the absence of canonical DNA fragmentation and thus activating mechanisms independent of caspase-3 but associated with different effector caspases, such as caspase-6 or -7 [166,167].

#### 3.5.3. Apoptotic-Related Protein Expression in Breast Cancer Cells

**Figure 12:**
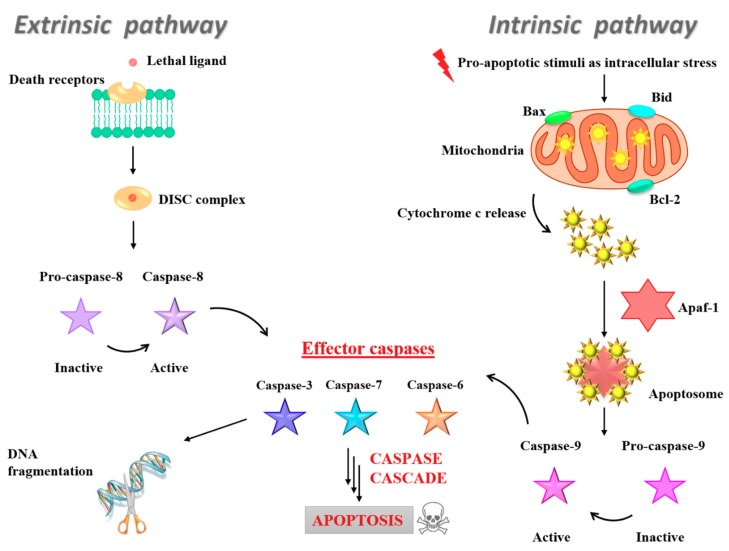
Schematic representation of the intrinsic (or mitochondrial) and the extrinsic (or death receptor) apoptotic pathways.

In addition to the fundamental role of caspase-3 in the DNA damage induction, all the intracellular caspases and BCL-2 (B-cell lymphoma-2) family proteins represent key elements for cancer response to chemotherapeutic intervention due to their involvement in the fine regulation of the apoptotic cell death machinery [168]. In particular, the family of BCL-2 contains protein factors playing a critical role in controlling dynamic cellular processes, decision-making between life and commitment to the mitochondrial apoptosis (intrinsic apoptotic pathway). BCL-2 was discovered as a new oncoprotein in acute lymphoblastic leukaemia, protecting cells from programmed cell death as apoptosis inhibitor and promoting cellular survival by inhibition of Bax and Bid action on mitochondria [169]. Many BCL-2 proteins with anti-apoptotic activities have been discovered, including B-cell lymphoma-extra-large (BCL-xL), BCL-2-like protein 2 (BCL-w), BCL-2-like protein 10 (BCL-B), myeloid cell leukemia 1 (MCL-1) and BCL-2 related gene A1 (A1). Conversely, the pro-apoptotic effectors of the BCL-2 family members, such as Bax and Bid, can commit a cell to its programmed death by promoting the permeabilization of its mitochondrial outer membrane (MOM) and the activation of the caspase cascade [170]. As a consequence, cyt*c* and other intermembrane space (IMS) mitochondrial proteins are released into the cytosol, playing a central role in the apoptosome formation that subsequently activates caspase 9. In turn, caspase 9 activates effector caspases 3, 6 and 7, cleaving vital cellular proteins and ultimately ensuring cellular demolition (Figure 12, right). The intrinsic pathway is typically activated by intracellular stress signals [165,171]. Several clinical studies demonstrated that the overexpression of the antiapoptotic BCL-2 protein is a negative prognostic marker in various tumours [172]; while high Bax expression was associated with a better response to chemotherapy in many cancers forms [173,174].

Conversely, the extrinsic pathway is generally activated by extracellular ligands able to bind to death receptors on the cell surface, leading to the formation of the death-inducing signalling complex (DISC). Formation of DISC—typically triggered by members of the death receptor superfamily, such as CD95 and tumour necrosis factor receptor—induces in turn caspase-8 activation and thereby the downstream caspase cascade (Figure 12, left) [175]. Thus, specific activation of some caspases or BCL-2 factors are suggestive of the induction of distinctive apoptotic pathways [171,175].

**Figure 13:**
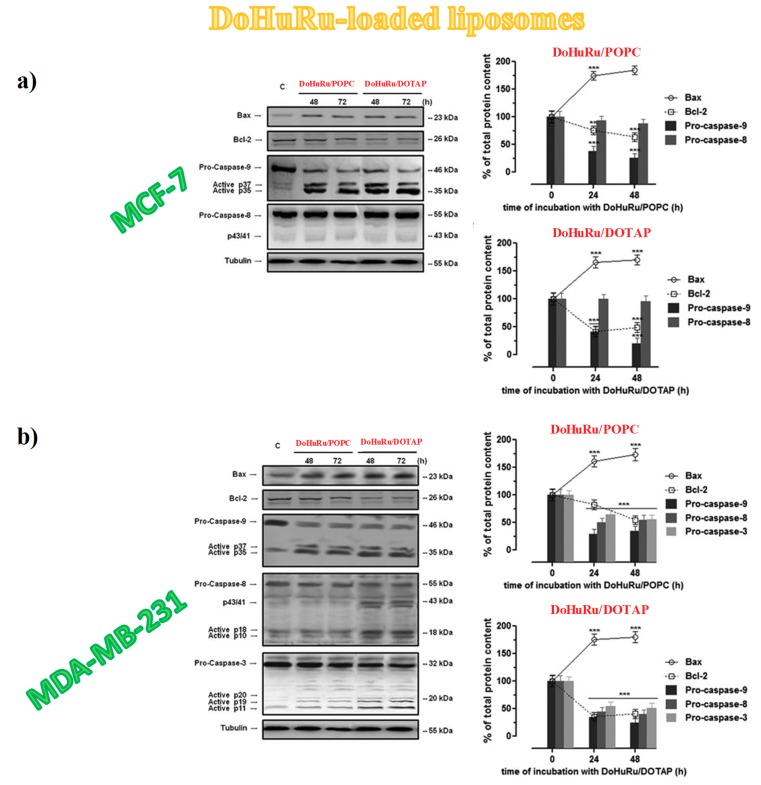
Western blot analysis showing the effects of IC_50_ doses of DoHuRu/POPC and DoHuRu/DOTAP following 48 and 72 h of incubation in MCF-7 (**a**) and MDA-MB-231 (**b**) cells on caspases-3, -8 and -9 expression and activation, and on Bax and BCL-2 expression. Bands resulting after chemoluminescence from treated cells were extracted, quantified by densitometric analysis and then plotted (graphs on the right of each panel): solid and dotted lines for Bax and BCL-2 expression proteins, respectively, and bar graphs (caspases-3, -8 and -9) reported as percentage of control in relation to the used Ru-containing nanoaggregates. Figures are adapted from [134].

Thus, Western blot analysis was used to investigate protein expression profile in treated MCF-7 and MDA-MB-231 cells after exposure to IC_50_ of DoHuRu/POPC and DoHuRu/DOTAP formulations at 48 and 72 h [134]. In both the investigated BCC lines, a considerable increase in caspase-9 activity with respect to untreated cells was detected—resulting in the production of active p35 and p37 subunits, known to amplify the apoptotic response—along with a simultaneous Bax up-regulation and BCL-2 down-regulation (Figure 13) [134]. These biological responses were time-dependent and more evident after treatment in vitro with the cationic DoHuRu/DOTAP liposome [134]. The activation of pro-caspase-9, together with the changed Bax/BCL-2 ratio, suggested a Ru-dependent activation of the apoptotic intrinsic pathway, probably as a result of interactions with putative mitochondrial targets, maybe via selective ROS generation and accumulation, as already found for other Ru(II) and Ru(III)-based drugs [176].

Notably, while no activation of caspase-8 appeared even after 72 h of exposure to the zwitterionic POPC-based nanosystems, the cationic DoHuRu/DOTAP nanoaggregates seemed to promote full length pro-caspase-8 cleavage, as evidenced by the formation of p10 and p18 fragments (Figure 13), fundamental active elements in the extrinsic death pathway [134].

Hence, DoHuRu/DOTAP formulations were able to concurrently trigger the induction of the two major apoptotic processes. Moreover, the activation of both apoptotic pathways (the intrinsic and the extrinsic one) seems to occur in a cell-specific manner, being evident only in MDA-MB-231 cells (Figure 13b). Possibly, the net positive surface charge of DOTAP-based nanoaggregates allows interacting in a peculiar manner with the cell membrane external bilayer, stimulating specific surface receptors involved in the activation of the death receptor pathway. However, these findings are not unprecedented since the activation of extrinsic apoptosis pathways [177], as well as the ability of some Ru(II) and Ru(III) complexes to simultaneously activate intrinsic and extrinsic apoptosis pathways, [178] have been demonstrated for other anticancer chemotherapeutics.

#### 3.5.4. Autophagy Activation in Breast Cancer Cells

In addition to apoptotic pathway, cellular suicide may also occur via non-apoptotic forms of programmed death such as autophagy [179,180]. Autophagic activity—generally low under basal conditions—can be specifically up-regulated in the presence of several physiological and non-physiological stimuli, including in vitro pharmacological treatments. [181,182] As in the case of apoptosis, autophagy play a crucial role in early stages of carcinogenesis, in cellular proliferation and survival [182,183,184,185,186,187,188]. Excessive autophagy is associated with cell death, as well as with other death pathways depending on cell types—an aspect to be taken into account as ruthenotherapy additionally activates apoptosis [189,190].

Since the activation of a pro-apoptotic response by chemotherapy can be affected by several factors, including cancer phenotypes and specific gene mutations, ultimately affecting the therapy success, the induction of alternative cell death pathway, alone or coupled to the apoptotic one [191], could represent an attractive goal in future anticancer intervention, especially in metastatic breast cancer cells [192,193]. The recent discovery of crosstalk connection among different cell death effectors and signalling pathways [194,195,196] will in fact offer novel effective therapeutic opportunities for targeted cancer therapies [197].

Notably, some Ru(II) complexes were found to be able to activate autophagy in cancer cells, though occasionally in antagonism to mitochondrial-mediated apoptosis [198,199]. A late step of the autophagic cell death consists of the autolysosomes formation, which is dependent on the coordinated activity of autophagy-related (Atg) proteins [186] involved in the maintenance of the cellular homeostasis [183,184,186,187,200]. Therefore, the formation of autophagic vacuoles visualized via monodansylcadaverine (MDC), a selective autofluorescent marker for autolysosomes detection [201]—in BCC treated with either DoHuRu/POPC or DoHuRu/DOTAP—was verified to evaluate the ability of Ru(III)-based compounds to induce also autophagy [134].

**Figure 14:**
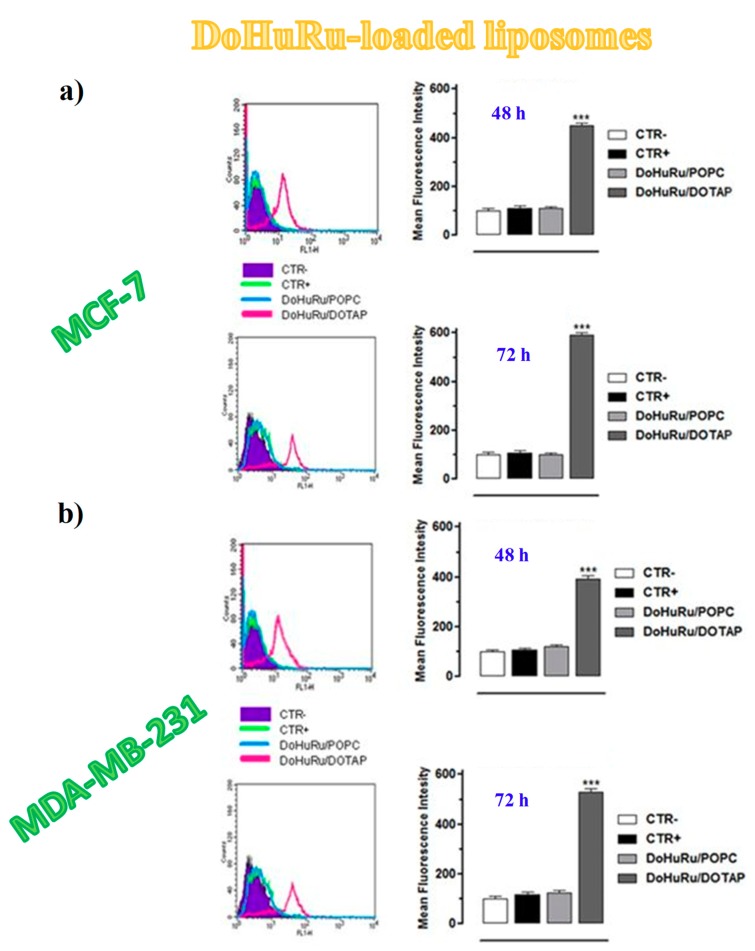
Quantitative flow cytometric analysis of autophagosomes formation (MDC incorporation) in MCF-7 (**a**) and MDA-MD-231(**b**) breast cancer cells for the autophagy activation evaluation: unlabelled and untreated (CTR −), labelled and untreated (CTR +), treated with IC_50_ concentrations of DoHuRu/POPC or with DoHuRu/DOTAP formulations for 48 and 72 h, as indicated. In the bar graphs, calculated main fluorescence intensities (MFIs) values are expressed as percentage of control cells. Figures are adapted from [134].

FACS analysis was used for the quantitative determination of MDC staining in MCF-7 and MDA-MB-231 cells exposed for 48 and 72 h to ruthenium IC_50_ doses (Figure 14) [134]. While the treatment with DoHuRu/POPC did not reveal significant autolysosome formation, the cellular exposure to DoHuRu/DOTAP liposomes indicated a significant formation of the MDC-labelled vacuoles already after 48 h (Figure 14). These results suggest the capability of the cationic nanoaggregates to activate autophagy in the presence of apoptosis [134].

The ability of DoHuRu-hosting cationic liposomes to induce autophagy could be associated with the ruthenium-induced down-regulation of the prosurvival protein BCL-2: indeed, interferences in the interaction between Beclin-1 and BCL-2 family proteins, by which Beclin-1 is inhibited in normal conditions, stimulate autophagy [202].

To get further insight into the ability of the Ru(III)-containing formulations to induce autophagic cell death in cancer cells, MCF-7 breast cells treated with HoThyRu/DOTAP liposomes were processed with a fluorescent autophagic detection kit able to measure autophagic vacuoles and to monitor the autophagic flux in live cells. DAPI was used to stain nuclei and FITC to visualize autophagic vesicles, such as autophagosomes and autophagolysosomes, and then the percentage of positive cells to autophagy was assessed [100]. In this way HoThyRu/DOTAP liposomes were demonstrated to strongly promote the formation of cytosolic autophagic vacuoles [100], reaching after 72 h similar effects to those induced by rapamycin, used as positive control being one of the most potent autophagy inducers [203].

#### 3.5.5. Autophagy-Related Proteins Expression in Breast Cancer Cells

**Figure 15:**
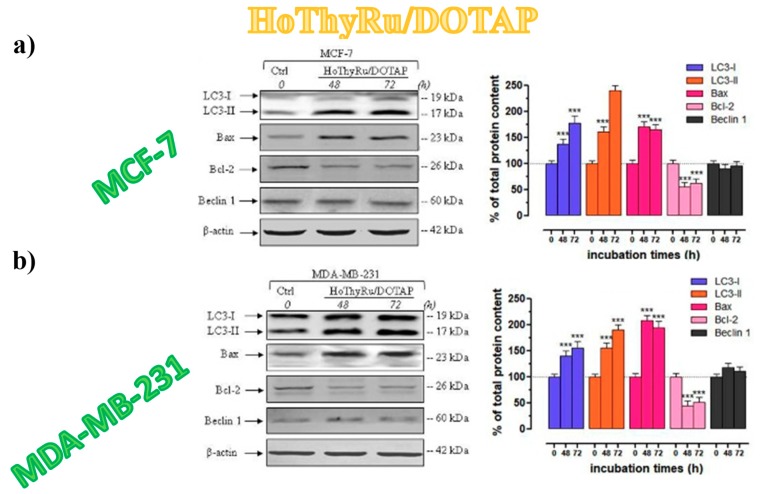
Western blot analysis showing the effects of IC_50_ doses of HoThyRu/DOTAP liposomes following 48 and 72 h of incubation in MCF-7 (**a**) and MDA-MB-231 cells (**b**) on LC3-I, LC3-II, Bax, BCL-2, and Becli-1 expression (Ctrl, untreated cells at the time zero). Bands resulting from MCF-7 and MDA-MB-231 cells were extracted, quantified by densitometric analysis and then plotted in bar graphs (right of each panel) as percentage of controls. Figures are adapted from [100].

The formation of autophagosomes represents the last step of autophagy process in which at least 16 proteins are involved: among these, only the LC3 protein strongly associates with the membrane of autophagosomes. This protein exists in two different forms: LC3-I and LC3-II, found respectively in the cytoplasm and as membrane-bound form. LC3-phosphatidylethanolamine conjugate (LC3-II) differs from LC3-I being covalently linked to lipid moieties [204] starting from LC3-I to initiate the formation of the autophagosome vacuoles [182,184]. The autophagosomal marker LC3-II reflects autophagic activity, so that LC3 monitoring by both immunoblotting and immunofluorescence has become a consistent method for autophagy detection [205]. In addition, the Beclin-1 activity is very important in autophagy cell death pathway: this protein is a component of the phosphatidylinositol-3-kinase (PI3K) complex, which is required for autophagosome formation, and the expression of Beclin-1 was found decreased in malignant breast epithelial cells [206,207,208]. Recent data also suggested a lack of autophagic pathways following Beclin-1 depletion which can be induced by different conditions, including chemotherapeutic interventions [209]. As demonstrated by immunoblot analysis (Figure 15), protein samples extracted from MCF-7 and MDA-MB-231 cells, following in vitro treatments with HoThyRu/DOTAP liposomes for 48 and 72 h, showed a relevant increase in both LC3 proteins, and mainly in the LC3-II form [100]. In parallel, Bax was significantly increased and BCL-2 decreased, and no relevant change between untreated and treated cells was found in Beclin-1 protein content (Figure 15).

As survival factor, BCL-2 down-regulation proved to promote the activation of autophagic cell death [210,211,212]. In addition, the maintenance of basal amounts of Beclin-1 after exposure to cationic Ru-based nanosystems could be decisive in regulating the induction of autophagy. Indeed, fluorescent bioimaging of MCF-7 cells treated with HoThyRu-containing DOTAP formulations and stained for LC3 indicated an intense protein immunoreactivity, likely localized into autophagic vacuoles in the cytoplasm [100].

### 3.6. In Vivo Anticancer Efficacy of Ru(III)-Containing Liposomes in Mice BCC Xenografts

**Figure 16:**
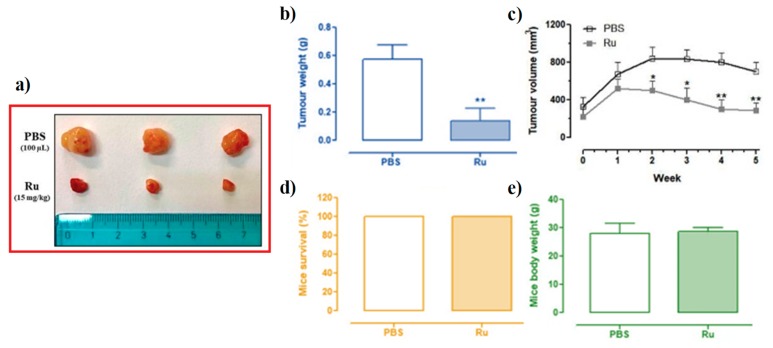
In vivo anticancer effects of HoThyRu/DOTAP nanosystems: tumour photographs after the collection (**a**); tumour weight (**b**) and tumour size (**c**) evaluation; mice survival (**d**) and body weight (**e**) determination (PBS control group, Ru treated group). Figures are adapted from [100].

In a preliminary investigation focused on in vivo response to HoThyRu/DOTAP formulation, athymic nude mice bearing human BCC xenografts were subcutaneously inoculated into the right flank with MCF-7 cells. Two weeks after the tumour growth, intraperitoneal injection of HoThyRu/DOTAP nanoaggregates was carried out once a week for four weeks. At the end of the schedule, the mice were sacrificed and the tumours collected (Figure 16) [100]. Subsequent analysis showed that the administration of HoThyRu/DOTAP formulation significantly inhibited breast cancer cell proliferation in mice, with a relevant reduction in both the weight and volume of tumour lesions with respect to the control group (Figure 16a–c). Notably, mice survival was 100%, and body weights were not affected by the ruthenium treatments (Figure 16d,e). Moreover, no evident toxicity was observed on the treated mice, suggesting a very good in vivo tolerance of the HoThyRu-hosting DOTAP nanosystems [100].

## 4. Improved Ru(III)-Containing Nanosystems: Introduction of Targeting and/or Diagnostic Agents for Theranostic Applications

Starting from the promising in vitro bioactivity of Ru(III)-containing POPC- and DOTAP-based liposomes, able to selectively and significantly reduce cancer cell survival, further improvements of these systems were recently proposed. In particular, two alternative approaches were reported based on varying the platform used to deliver the nucleolipid-based Ru(III)-complexes.

In a first approach, niosome vesicles were used to replace liposomes.

In detail, vesicles prepared from non-ionic surfactants [213,214], currently known as niosomes or NSVs (non-ionic surfactant vesicles), gained increasing attention as drug delivery systems, providing a cheaper and more stable alternative to phospholipids used in liposome preparation [215,216]. Niosomes are self-assembled vesicles made up of single chain non-ionic surfactants combined with cholesterol or other lipids [217,218]. Charged molecules can be also added as ingredients to better stabilize the obtained formulations and prevent their aggregation over time [214,215,218,219].

Owing to their vesicle structure, niosomes are able to incorporate both hydrophilic and hydrophobic drugs, just like liposomes do [216,218,220,221]. The drug is entrapped in a membrane resulting from the self-assembly of the surfactant + lipid molecules, generally organized in stable bilayers [222]. Niosomes can protect the loaded drugs from premature degradation or possible inactivation overcoming some major biopharmaceutical problems such as insolubility, side effects, and poor chemical stability of drugs [223,224]. Moreover, the high chemical stability of surfactants, compared with phospholipids, makes niosome purification, handling and storage much easier than those of conventional liposomes [225,226].

In our approach, the use of niosomal formulations allowed the introduction in the lipid formulation, besides the Ru(III)-based complexes, also of an active targeting agent, i.e., the oligodeoxyribonucleotide AS1411 [109]. This G-rich 26-mer, carrying the sequence ^5′^GGTGGTGGTGGTTGTGGTGGTGGTGG^3′^, entered in Phase II anticancer clinical trials, selectively targets nucleolin, a multifunctional protein involved in cell survival, growth and proliferation, overexpressed on the outer membrane of cancer cells (for a recent review covering the state-of-the-art knowledge on AS1411, see P. J. Bates et al. [227]).

Recently, AS1411 has been largely employed as a selective nucleolin-targeting agent, successfully combined with chemotherapeutic agents (such as paclitaxel [228,229,230,231], docetaxel [232,233,234], doxorubicin [235,236,237,238,239,240,241,242,243,244], and epirubicin) [245] onto suitable nanosystems in the context of specific recognition of cancer cells and tumour-selective delivery of therapeutic or imaging agents for cancer treatment [227].

In the proposed niosome formulations, AS1411 was for the first time associated with a Ru(III)-based chemotherapeutic drug to improve its selective delivery via specific recognition of cancer cells [109].

In a second approach, superparamagnetic iron oxide NPs (SPIONs) with a core/double shell architecture were exploited as platform for Ru incorporation, proving to be effective contrast agents for in vivo Magnetic Resonance Imaging (MRI) investigations and thus potential tools in theranostic nanomedicine, intended to provide simultaneous diagnosis and treatment of a disease, with early detection, easy monitoring, targeted therapy and minimal toxicity [246,247].

### 4.1. Niosome-Based Systems Containing Nucleolipid-Based Ru(III) Complexes

**Figure 17:**
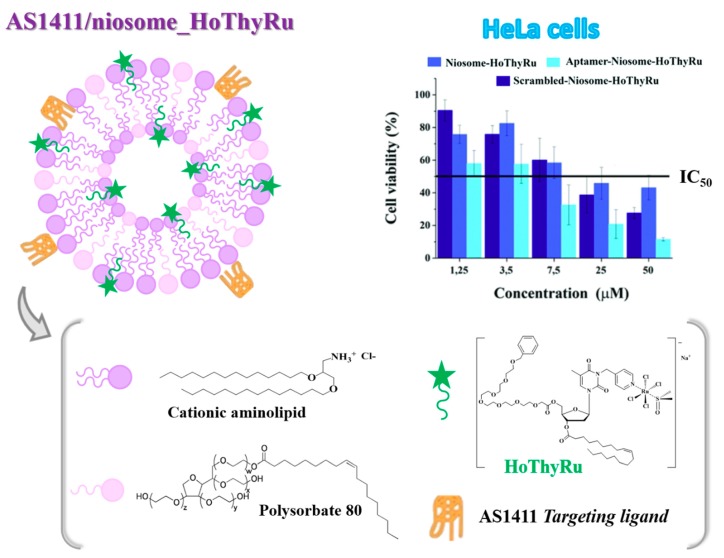
Schematic representation of niosome formulations based on a cationic aminolipid and the polysorbate 80, also containing the nucleolipid-based Ru(III) complex HoThyRu along with AS1411. On the right, the concentration/effects bar graphs obtained after in vitro treatment on HeLa cancer cells were also reported as representative examples. Figures were adapted from [109].

With the aim of exploring niosome vesicles as versatile scaffolds for effective loading of multiple anticancer drugs to be used in combination therapies, Riccardi et al. [109] devised a novel formulation—mixing a cationic lipid, i.e., [2,3-di(tetradecyloxy)propan-1-aminium chloride] and the non-ionic surfactant polysorbate 80 in 3:1 molar ratio—as optimized vesicles with respect to previously described ones (Figure 17) [248,249]. Then, these niosomal formulations were decorated with the anticancer drug HoThyRu—hitherto one of the most effective ones among the nucleolipidic-based Ru(III) complexes investigated—as well as with the G-quadruplex-forming aptamer AS1411, to ensure highly specific recognition towards cancer cells (Figure 17) [109].

The morphology, average size, zeta potential, electrophoretic mobility and stability over time of the functionalized niosomes were analysed using different biophysical techniques.

In particular, both unfunctionalized and functionalized niosomes gave monodisperse vesicle-like aggregates, with typical size of ca. 100 nm, stable upon storage for one month, also able to fully incorporate both the nucleolipid Ru(III) complex HoThyRu and the oligonucleotide AS1411. The functionalization was driven by electrostatic interactions, in the case of the negatively-charged oligonucleotide, and by a combination of hydrophobic and electrostatic interactions for HoThyRu encapsulation [109].

Then, the antiproliferative activity of the niosome formulations was evaluated using the MTT assay on a selected panel of human cells including a human cervix adenocarcinoma (HeLa) and two colorectal cancer cell lines (HTB-38, HCC2998), as well as a non tumoural embriogenic human cell line (HEK293T). HeLa cells were selected as a relatively drug-sensitive cell line with high nucleolin content, already proved to be responsive to niosome formulations [249] as well as to AS1411 [227]. In contrast, HTB-38 and HCC2998 colorectal cancer cell lines were chosen being highly resistant even to very effective chemotherapeutic agents such as 5-fluorouracil and camptothecin [250,251]. Finally, the embryogenic cell line HEK293T was included as healthy cell line, already investigated to verify the absence of toxicity of niosome formulations [252].

Niosome formulations containing both AS1411 and HoThyRu showed increased bioactivity compared to those carrying only HoThyRu, proving the ability of the nucleolin-targeting aptamer to markedly enhance the bioactivity of the Ru(III)-containing niosomes (in Figure 17, on the right, the concentration/effect curves obtained on HeLa cells were reported as a representative example).

The obtained response was however cell-dependent, with HeLa cells more sensitive to this drug treatment than colorectal cancer cells [109]. The observed lower antiproliferative activity on both HCC2998 and HTB-38 colorectal cancer cell lines can be explained considering their generally poor sensitivity to drug treatments and low expression of nucleolin. Taken together, these results proved niosome formulations as very interesting nanocarriers to transport in cells anticancer drugs and the efficacy of AS1411 as targeting agent also in niosomal systems [109].

### 4.2. Nanoparticle-Based Systems Containing Nucleolipid-Based Ru(III) Complexes

Stable phosphocholine-functionalized SPIONs (18LPC/SPIONs) were recently prepared through thermal decomposition method exploiting hydrophobic interactions between a suitable phosphocholine, i.e., 1-octadecyl- 2-hydroxy-*sn*-glycero-3-phosphocholine (18LPC), and an inner amphiphilic layer (composed by oleic acid and oleylamine), covering the surface of the NPs [108,253,254].

**Figure 18:**
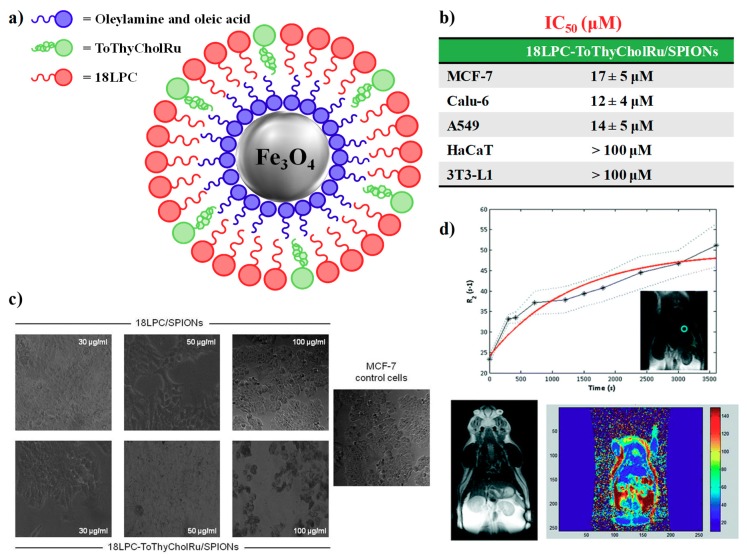
18LPC-ToThyCholRu/SPIONs: schematic representation of NPs systems with the inner membrane layer based on oleylamine/oleic acid and the outer membrane layer composed of 18-LPC loaded with the Ru(III)-complex ToThyCholRu (**a**); IC_50_ values (μM)—reported as mean ± SEM—relative to the effective ruthenium concentration carried by 18LPC-ToThyCholRu/SPIONs in the indicated cell lines following 48 h incubation (**b**); representative photomicrographs by phase-contrast light microscopy (**c**) of MCF-7 cell lines untreated (control cells, left) or treated for 48 h with the indicated iron concentration of 18LPC/SPIONs (top line) and of 18LPC-ToThyCholRu/SPIONs (bottom line); MRI measurements (**d**): transverse relaxation rate (R_2_ = 1/T_2_, s^−1^) measured after bolus injection in the tail vein of 18LPC/SPIONs in 0.9% NaCl solution (top) with inset representing the ROI, selected in the coronal plane covering the liver; T_2_-weighted image of a coronal view of the animal (Wistar rat) before injection of the 18LPC/SPIONs (bottom, left); representative T_2_-mapping MRI image of a coronal view after injection (t = 3 min) of the 18LPC/SPIONs (bottom, right). Figures are adapted from [108].

18LPC-SPIONs were further decorated with the nucleolipid-based Ru(III)-complex ToThyCholRu (18LPC-ToThyCholRu/SPIONs) [108], lodged in the external coating layer of the nanoparticle surface through reversible hydrophobic interactions (Figure 18a). Then, all the prepared SPION systems were characterized by DLS, TEM, small-angle X-ray scattering (SAXS) and wide-angle X-ray scattering (WAXS) measurements.

The successive evaluation of the bioactivity of the ToThyCholRu-functionalized SPIONs on selected human cancer and non-cancer cells (MCF-7 breast adenocarcinoma, human HaCaT keratinocytes, murine 3T3L-1 fibroblasts, human Calu-6 and A549 lung carcinoma cell lines) confirmed the potential of this system as an effective theranostic device (Figure 18b) [108]. Indeed, 18LPC/SPIONs were devoid of significant biological effects, even at 100 μg/mL iron concentration on both cancer and healthy cell lines, (Figure 18b) while the incorporation of the amphiphilic ruthenium complex ToThyCholRu in the SPION surface induced a significantly enhanced bioactivity, providing significant cytotoxic effects in cancer cells without affecting healthy cells (Figure 18b) [108]. Notably, when inserted in the SPION nanosystems, ToThyCholRu was also very effective in inhibiting MCF-7 proliferation, with ca. four-fold higher cytotoxicity compared to the same Ru(III) complex loaded in POPC; conversely the inhibition efficiency was similar to that observed after loading in cationic DOTAP-based nanoaggregates (see data reported in Table 1).

In analogy to previous investigations (Section 3.4), subconfluent breast MCF-7 culture cells were also examined by phase-contrast light microscopy indicating that the in vitro exposure to 18LPC/SPIONs did not alter cell morphology, whereas after Ru-loaded NPs administration, significant cytological changes of the cell monolayers clearly appeared (Figure 18c) [108].

Once assessed for their biocompatibility, 18LPC/SPIONs were also injected in the tail vein of a healthy rat, chosen as a small animal model to evaluate their in vivo pharmacokinetics and applicability as negative contrast agents for MRI analyses (Figure 18d). This effect is typically evaluated with the transverse proton relaxation time (T_2_) reduction or transverse proton relaxation rate (R_2_) increase [255,256].

Ten minutes after injection, a notable T_2_ relaxation time decrease was recorded in the liver of the animal, consistently with the nanoparticle capture by the reticuloendothelial hepatic tissue (Kupffer cells) [257], without significant variation in other organs (e.g., kidneys and heart). Then, according to the progressive accumulation of the 18LPC/SPIONs in the tissue for excretion, R_2_ reached a maximum value 60 min after injection [108].

Thus, 18LPC/SPIONs demonstrated to favour long circulation times with a measurable reduction in the MR signals in the rat liver, indicating a very promising potential for use as MRI negative contrast agents [108].

More recently, as additional ingredients for the SPIONs surface decoration, suitable lipophilic AS1411 conjugates were also studied [258] in order to evaluate in future studies the ability of AS1411 to enhance the Ru(III)-based compounds uptake and, thus, their overall anticancer activity also when incorporated onto SPIONs.

## 5. Conclusions

Cancer is one of the leading causes of death in the world, representing a key topic in scientific research. Cancer cells are able to accumulate multiple mutations allowing their uncontrollable growing, invasiveness, and eventually spreading to other parts of the body causing tumour metastasis formation. In addition, malignant cells typically exhibit aberrations in the physiological signalling pathways regulating various cellular processes, such as cell growth and division, as well as cell-to-cell communication. Therefore, the genetic and phenotypic heterogeneities of tumours form, making cancer a very difficult disease to treat.

Stimulated by the success of cisplatin, intense efforts have been devoted to the development of alternative transition metal-based anti-tumour compounds. Pioneering studies in this field led to promising lead Ru(III)-complexes, such as NAMI-A, KP-1019 and NKP-1339, which entered clinical trials, even though none of these have been approved yet for human use.

The difficulty of progressing lead compounds from animal studies to human clinical trials and/or effective clinical use highlights the complications involved in overcoming the pharmacokinetic and pharmacodynamic barriers in living animals. Metal compounds with proved selective anti-tumour in vitro activity are presumably able to reach the target tumour xenograft without causing adverse effects to the health of the animal. Nevertheless, this is not always verified since a compound active in vitro against cancer cell lines can fail under in vivo testing for several reasons. For example, the lipophilicity of complexes is determinant for effective cellular uptake. Secondly, the compound may be too unstable in human plasma or may be cleared too quickly by the liver. Alternatively, a compound may be too toxic to a particular organ, even though it may not show cytotoxicity in vitro against normal cell lines.

In this context, the encapsulation of Ru(III) complexes into nanostructures is an increasing research area, particularly useful to address the uptake and stability issues of these anticancer agents; therefore, some ground-breaking advances are expected in the near future. Nanoformulations can be used to enhance the efficacy of Ru complexes in cancer treatment by targeted delivery of drugs into cancer cells and reduce their adverse effects and systemic toxicity by optimization of their pharmacokinetic properties. Notably, examples have been here discussed showing the ability of Ru(III)-functionalized liposomes to activate multiple cell death pathways, which represents a very interesting perspective in order to develop new intervention strategies in TNBC with limited chemotherapeutic options.

Although nanostructured materials functionalized with Ru complexes (micelles, liposomes, niosomes and iron oxide nanoparticles) have shown great potential in the treatment of cancer, there are still many challenges in translating basic research into clinical applications.

To reach these challenging goals, understanding the pharmacokinetic and pharmacodynamic parameters of a drug is essential for the design of dosage administration regimens, as well as to help reducing toxicity effects and enhancing target drug delivery. A more in-depth understanding of the mechanism of action of the hit/lead compounds could also potentially help to improve the potency and selectivity of the candidate drugs through rational design of optimized drugs.

Finally, significant efforts must be invested in developing extensive in vivo screenings in models that mimic human pathophysiology in order to achieve higher predictability for successful Ru(III)-drug development.

Overall, research on the encapsulation of Ru(III) complexes will undoubtedly continue to develop and, owing to the high structural variety of nanosystems and Ru derivatives, new delivery platforms will certainly be realized in the near future for anticancer therapies.

Based on the continuous efforts that worldwide researchers have invested in this field, our feeling is that it is only a matter of time before new metal complexes with superior efficacy and low toxicity will be approved as anticancer therapeutics, finally replacing longstanding metal-based anticancer drugs as cisplatin and its derivatives.

In this context, the next years will witness progress in elucidating real targets and mechanisms of action that will provide solid scientific basis for extensive preclinical development of selected Ru(III)-containing formulations.

## Figures and Tables

**Table 1:** IC_50_ values (µM) relative to AziRu and to the effective ruthenium concentration inserted in the Ru(III)-incorporating POPC- and DOTAP-based liposomes in the indicated cell lines following 48 or 72 h of incubation in vitro. IC_50_ values are reported as mean ± SEM.

IC_50_ (μM)						
	MCF-7	WiDr	C6	HeLa	L6	HaCaT
NAMI-A [78]	620 ± 30	-	-	626 ± 45	-	-
AziRu [77,79]	305 ± 16 [77]	441 ± 20 [77]	318 ± 12 [77]	382 ± 19 [79]	> 500 [79]	> 500 [79]
POPC-based formulations						
ToThyRu/POPC [77,134]	27.8 ± 0.1 [134]	75 ± 4 [77]	36 ± 8 [77]	-	-	-
HoThyRu/POPC [77]	7 ± 4	40 ± 5	81 ± 7	-	-	-
DoHuRu/POPC [77,134]	18.9 ± 0.1 [134]	99 ± 5 [77]	24 ± 5 [77]	-	-	-
ToThyCholRu/POPC [79,105]	70 ± 12 [105]	165 ± 10 [105]	-	-	>500 [79]	>500 [79]
HoUrRu/POPC [107]	14 ± 7	20 ± 8	-	-	-	-
DOTAP-based formulations						
ToThyRu/DOTAP [106,134]	10.1 ± 0.1 [134]	50 ± 11 [106]	54 ± 8 [106]	-	-	-
HoThyRu/DOTAP [100,106]	13 ± 4 [100]	65 ± 8 [106]	43 ± 11 [106]	-	-	-
DoHuRu/DOTAP [106,134]	10.3 ± 0.2 [134]	41 ± 10 [106]	34 ± 9 [106]	-	-	-
ToThyCholRu/DOTAP [79]	13 ± 2	23 ± 8	-	34 ± 4	187 ± 1	377 ± 3
HoUrRu/DOTAP [107]	8 ± 5	12 ± 5	-	-	-	-
I/DOTAP [110]	31.2 ± 2.7	-	35.4 ± 2.7	45.6 ± 3	-	>150

**Table 1:** Host range and plaque morphology of the 19 phages examined in this paper. Phages were isolated from various environments: natural springs, industrial waste water and chicken meat. The infectivity of the phages was calculated based on the host range (Figure 1). Phages exhibited various plaque morphologies, varying from very small (less than 0.5 mm diameter) to large (up to 4 mm diameter). Plaques were formed on agar plates with *E. coli *host that was used to isolate the phages.

	The Composite Sustainability Indicator (CSI)						Average Score^①^	Growth Rate^②^
	2011	2012	2013	2014	2015	2016		
Beijing	0.565	0.59	0.609	0.605	0.588	0.634	0.598	2.44%
Tianjin	0.562	0.555	0.553	0.513	0.522	0.515	0.537	−1.66%
Hebei	0.403	0.401	0.414	0.426	0.467	0.503	0.436	4.93%
Shanxi	0.407	0.419	0.442	0.434	0.437	0.482	0.437	3.70%
Inner Mongolia	0.522	0.523	0.532	0.556	0.558	0.598	0.548	2.89%
Liaoning	0.474	0.486	0.515	0.509	0.509	0.453	0.491	−0.91%
Jilin	0.406	0.442	0.455	0.466	0.467	0.517	0.459	5.46%
Heilongjiang	0.393	0.397	0.432	0.431	0.432	0.46	0.424	3.42%
Shanghai	0.45	0.455	0.449	0.477	0.496	0.556	0.48	4.69%
Jiangsu	0.53	0.517	0.555	0.547	0.566	0.586	0.55	2.11%
Zhejiang	0.534	0.545	0.576	0.588	0.594	0.613	0.575	2.96%
Anhui	0.412	0.43	0.503	0.483	0.51	0.582	0.486	8.25%
Fujian	0.487	0.525	0.537	0.547	0.574	0.586	0.543	4.03%
Jiangxi	0.403	0.42	0.45	0.423	0.45	0.507	0.442	5.20%
Shandong	0.537	0.537	0.572	0.574	0.561	0.588	0.561	1.90%
Henan	0.33	0.337	0.351	0.382	0.402	0.484	0.381	9.30%
Hubei	0.421	0.446	0.458	0.501	0.518	0.574	0.486	7.27%
Hunan	0.398	0.431	0.433	0.464	0.508	0.551	0.464	7.70%
Guangdong	0.474	0.451	0.482	0.471	0.514	0.534	0.488	2.54%
Guangxi	0.415	0.431	0.436	0.443	0.477	0.522	0.454	5.18%
Hainan	0.547	0.596	0.595	0.585	0.569	0.598	0.582	1.87%
Chongqing	0.496	0.531	0.541	0.571	0.572	0.604	0.553	4.36%
Sichuan	0.388	0.401	0.407	0.417	0.46	0.512	0.431	6.41%
Guizhou	0.393	0.427	0.422	0.452	0.473	0.518	0.448	6.35%
Yunnan	0.427	0.432	0.446	0.457	0.449	0.504	0.452	3.58%
Tibet	0.534	0.55	0.56	0.582	0.582	0.558	0.561	0.92%
Shaanxi	0.483	0.521	0.532	0.526	0.545	0.568	0.529	3.53%
Gansu	0.421	0.429	0.442	0.442	0.43	0.474	0.44	2.49%
Qinghai	0.476	0.485	0.49	0.504	0.502	0.549	0.501	3.06%
Ningxia	0.457	0.481	0.501	0.512	0.503	0.551	0.501	4.15%
Xinjiang	0.5	0.494	0.498	0.515	0.546	0.567	0.52	2.68%
East China^③^	0.509	0.517	0.534	0.533	0.545	0.571	0.535	2.45%
Middle China	0.395	0.414	0.439	0.448	0.471	0.53	0.449	6.83%
West China	0.459	0.475	0.484	0.498	0.508	0.544	0.495	3.68%
Northeastern China	0.424	0.442	0.467	0.469	0.469	0.477	0.458	2.46%
